# Novel Insights into the Thioesterolytic Activity of *N*-Substituted Pyridinium-4-oximes †

**DOI:** 10.3390/molecules25102385

**Published:** 2020-05-21

**Authors:** Blaženka Foretić, Vladimir Damjanović, Robert Vianello, Igor Picek

**Affiliations:** 1 Department of Chemistry and Biochemistry, School of Medicine, University of Zagreb, Šalata 3, HR-10000 Zagreb, Croatia; vladimir.damjanovic@mef.hr; 2 Division of Organic Chemistry and Biochemistry, Ruđer Bošković Institute, Bijenička 54, HR-10000 Zagreb, Croatia; Robert.Vianello@irb.hr

**Keywords:** acetylthiocholine, pyridinium-4-oxime-ester, reaction mechanisms, energy profiles, computations

## Abstract

The pyridinium oximes are known esterolytic agents, usually classified in the literature as catalysts, which mimic the catalytic mode of hydrolases. Herein, we combined kinetic and computational studies of the pyridinium-4-oxime-mediated acetylthiocholine (AcSCh^+^) hydrolysis to provide novel insights into their potential catalytic activity. The *N*-methyl- and *N*-benzylpyridinium-4-oximes have been tested as oximolytic agents toward the AcSCh^+^, while the newly synthesized *O*-acetyl-*N*-methylpyridinium-4-oxime iodide was employed for studying the consecutive hydrolytic reaction. The relevance of the AcSCh^+^ hydrolysis as a competitive reaction to AcSCh^+^ oximolysis was also investigated. The reactions were independently studied spectrophotometrically and rate constants, *k*_oxime_, *k*_w_ and *k*_OH_, were evaluated over a convenient pH-range at *I* = 0.1 M and 25 °C. The catalytic action of pyridinium-4-oximes comprises two successive stages, acetylation (oximolysis) and deacetylation stage (pyridinium-4-oxime-ester hydrolysis), the latter being crucial for understanding the whole catalytic cycle. The complete mechanism is presented by the free energy reaction profiles obtained with (CPCM)/M06–2X/6–311++G(2df,2pd)//(CPCM)/M06–2X/6–31+G(d) computational model. The comparison of the observed rates of AcSCh^+^ oximolytic cleavage and both competitive AcSCh^+^ and consecutive pyridinium-4-oxime-ester hydrolytic cleavage revealed that the pyridinium-4-oximes cannot be classified as non-enzyme catalyst of the AcSCh^+^ hydrolysis but as the very effective esterolytic agents.

## 1. Introduction

The mono- and bis-pyridinium oximes, as quaternized derivatives of pyridyl oximes, are known as pharmacologically important agents [1]. Explicitly, the deprotonation of the oxime group produces the oximate form, >C=N-O^−^, a powerful nucleophile used in the cleavage of either phosphoric and carboxylic acid esters or amides. A nitrogen atom with an unshared electron pair adjacent to the oxy-anionic nucleophilic center makes pyridinium-4-oximes α-nucleophiles that exhibit a much higher reactivity than common oxygen nucleophiles of similar basicity. The ability of oximes to act as esterolytic agents had already been recognized in the late 1950s and various terms like oximolysis, esterolysis, thiocholine ester hydrolysis and cholinesterase pseudo-activity have been adopted to describe the phenomenon [2,3,4,5,6,7,8,9,10]. This reflects their biological role, making them especially effective reagents involved in the reactivation of acetylcholinesterase (AcChE) initially inhibited by organophosphorus poisons (pesticides, nerve gases) [1] or catalysts, which mimic the catalytic mode of hydrolases, as well as excellent Michael donors in nucleophilic additions [11]. The biological importance and pharmacological application of pyridinuim oximes arises from their abilities to reactivate AcChE, the essential enzyme involved in neurotransmission, inhibited by organophosphorus poisons. The phosphylation of serine hydroxyl group in the active site of AcChE results in the accumulation of acetylcholine (AcCh^+^) in the synaptic clefts, leading to overstimulation of cholinergic receptor sites. Even though the antidotal potency of pyridinium oximes is primarily attributed to their ability to displace the phosphoryl moiety from the enzyme active site by virtue of their powerful nucleophilicity, they can also bind to AcChE either at the active site or at the allosteric site acting as reversible inhibitors, thus explaining their mechanism of AcChE protection. In addition, so-called direct pharmacological effects such as direct reaction with organophosphorus nerve poisons, anticholinergic effect and properties that decrease the amount of liberated AcCh^+^ into synaptic cleft are also known [1]. The in vitro AcChE binding and reactivation capability of novel lipophilic pyridinium oxime derivatives and their structural analogues are still extensively studied in a search for universal antidote capable to cross the blood-brain barrier [12,13]. However, these pharmacological compounds possess multiple modes of action and alternative bioactivities are an active research topic. A particular focus is placed on electron and charge transfer processes where pyridinium-4-oxime cations are recognized as new electron acceptors for the formation of colored, supramolecular, inter-ionic charge-transfer complexes with hexacyanoferrate(II) anion [14].

Beside the ubiquity of thioesters in living systems playing central roles in metabolism, the use of acetylthiocholine (AcSCh^+^), the O→S substituted analogue of the AcChE natural substrate, AcCh^+^, dates from the mid-twentieth century. A standard spectrophotometric protocol utilizing AcSCh^+^, developed by Ellman, is used in various assays for probing the presence and activity of AcChE in biological fluids, cell suspensions, tissues and so forth [15]. An alternative to AcChE use in biosensing approaches comprises the application of bioinspired AcChE-based models in which pseudo AcChE-molecules promote hydrolysis of AcCh^+^ or AcSCh^+^ and organophosphorus esters or carbamates [16]. The carboxylic acid thio- and oxy-esters are rapidly cleaved during metabolism by hydrolytic enzymes that catalyze either direct water attack on the ester linkage or a double-displacement reaction in which the enzyme and the substrate form an acyl-enzyme intermediate that subsequently undergoes hydrolysis (such as AcChE) [17]. Non-enzymatic hydrolysis of esters containing the acetyl group, beside the nucleophilic attack by H_2_O, termed neutral or “water” (or pH-independent or uncatalyzed) hydrolysis comprises acid and base catalyzed hydrolysis. In the acid catalyzed mechanism, that is, catalysis by H^+^, if the proton source is hydronium ion (H_3_O^+^), (and the ester is already protonated in the rate-limiting step of the reaction) the catalysis is termed specific acid catalysis. Specific base catalysis by HO^−^ ions is similar in that the base is hydroxide and the ester is attacked by hydroxide ion in the rate-limiting step of the reaction (there are no other bases, for example, such as the conjugate base of an acid, in the rate-limiting step). Each of these processes occurs independently of the others. Catalysis also occurs when an undissociated acid is present in the rate-limiting step of the reaction. Such a catalysis is termed general acid catalysis and the transfer of the proton to the substrate occurs in the rate-limiting step of the mechanism. Catalysis that involves the conjugate base of an acid in the slow step of the reaction is termed general base catalysis. In this mechanism the conjugate base of the acid deprotonates water, which simultaneously attacks the substrate. The process is often called general base assisted nucleophilic attack. Kinetics and mechanism of carboxylic acid esters hydrolysis are generally well understood including the influence of both, electronic and steric effects on their reactivity [18,19,20,21]. AcCh^+^ is more susceptible to neutral and base-catalyzed hydrolysis than ethyl acetate but less susceptible to acid-catalyzed hydrolysis [8], while majority of thioesters undergoes both acid and base-catalyzed hydrolysis [10]. Kinetic studies indicated that there are two distinct types of thioesters—one which possesses an activated carbonyl group due to the presence of electron-withdrawing substituents on the α-carbon and second type which lacks an activated carbonyl—unactivated thioesters such as AcSCh^+^ [11]. Unactivated thioesters display three distinct regions in the pH-rate profile—HO^−^-ion catalysis above pH 7, neutral (uncatalyzed) hydrolysis between pH 2–7 and H^+^-ion catalysis below pH 2 [11].

The esterolytic potencies of pyridinium oximes in the inhibited, usually phosphylated, AcChE reactivation kinetics or in the non-enzymatic AcSCh^+^ hydrolysis reaction were widely studied [8,9,22,23]. In these pyridinium oxime-(thio/phospho)ester reactions, an oximate anion, as a nucleophile, attacks the carbonyl carbon or phosphorus atom of the (thio/phospho)ester, respectively, promoting the acylation/phosphylation of the pyridinium oxime. Certainly, in a competing process thioester slowly spontaneously hydrolyzes in water (*t*_1/2_ ≈ 210 min at 24 °C, *k*_w_ = 3.3 × 10^−3^ min^−1^ for unactivated thioesters [19]), while phosphylation of the active site serine in the inhibited AcChE results in the uncharged adduct which is unreactive toward spontaneous hydrolysis [23]. More importantly, the further fate, structural and energetic features of the produced pyridinium oxime-ester (specifically acetylated pyridinium oximes formed in the reaction with AcSCh^+^) is crucial for understanding the whole thermodynamic and catalytic cycle but usually undeveloped in the available literature.

**Scheme 1:**
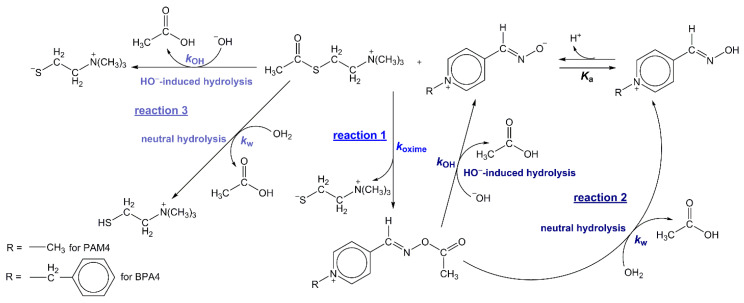
The fate of AcSCh^+^ in the aqueous reaction system that contains various nucleophiles.

To provide insights into the role of pyridinium oximes such as pseudo-hydrolases (thioesterases), two well-defined pyridinium-4-oximes, *N*-methylpyridinium-4-oxime iodide (PAM4^+^I^−^) and *N*-benzylpyridinium-4-oxime chloride (BPA4^+^Cl^−^) [14,24], have been tested as oximolytic agents toward the AcSCh^+^. To complete the cycle of the pyridinium-4-oximate promoted reactions, the hydrolytic susceptibility of the newly synthetized pyridinium-4-oxime-ester, *O*-acetyl-*N*-methylpyridinium-4-oxime iodide (AcPAM4^+^I^−^), has been separately investigated. Herein, we report for the first time the synthetic pathway and isolation of this novel oxime-ester, along with its physico-chemical characterization. The combined kinetic and computational mechanistic studies were undertaken and relevant kinetic and thermodynamic parameters revealed. The investigated reaction system (Scheme 1) included three pH-dependent reactions—(1) the oxime-thioester reaction between oximate ion and AcSCh^+^, that is, oxime-assisted thioesterolysis of AcSCh^+^—termed oximolysis; (2) the consecutive neutral or HO^−^-catalyzed hydrolysis of pyridinium-4-oxime ester (AcOxime^+^) formed by oximolysis and (3) the competitive neutral or HO^−^-catalyzed AcSCh^+^ hydrolysis.

## 2. Results and Discussion

The investigated reaction system is presented on Scheme 1. The reactions 1, 2 and 3 were independently studied spectrophotometrically and rate constants, *k*_oxime_, *k*_w_ and *k*_OH_ were evaluated over a convenient range of pH at *I* = 0.1 M and 25 °C. In addition, the density functional theory (DFT) computational analysis provided a mechanistic insight into these reactions along with kinetic $\left(\Delta G_{\mathrm{COMP}}^{‡}\right)$ and thermodynamic (Δ*G*_R COMP_) parameters.

The nucleophilic ability of the examined pyridinium-4-oximes to promote AcSCh^+^ esterolysis was investigated by measuring the rates of reactions between individual pyridinium-4-oxime and AcSCh^+^ at different pH. In each set of experiments, the pH was kept constant by means of an external buffer (*I* = 0.1 M, 25 °C) and the concentration of the reactive deprotonated pyridinium-4-oxime (oximate) was defined according to the ionization equilibrium (Scheme 1) as:

(1) $$\left[\mathrm{oximate}\right]=c\left(\mathrm{oxime}\right)\cdot \frac{1}{10^{pK_{a}-\mathrm{pH}}+1}$$

To determine second-order rate constant *k*_oxime_ for oximolysis over a convenient range of pH, the pseudo-first-order rate constants, *k*_obs_, were evaluated. In a series of experiments, conducted with a large molar excess of AcSCh^+^ over the concentration of the oximate, the rates of disappearance of oximate ions were followed at the wavelength of the absorption maximum of the reactive oximate (see experimental for details). The application of the derivated rate law to reaction 1 gives the Equation (2):

(2) $$-\frac{d\left[\mathrm{oximate}\right]}{dt}=k_{\mathrm{oxime}}\cdot \left[\mathrm{oximate}\right]\cdot \left[{\mathrm{AcSCh}}^{+}\right]=k_{\mathrm{obs},\mathrm{oximolysis}}\cdot c\left(\mathrm{oxime}\right)$$

Accordingly, the combination of Equations (1) and (2) results in the following expression:

(3) $$k_{\mathrm{obs},\mathrm{oximolysis}}=k_{\mathrm{oxime}}\cdot \frac{1}{10^{pK_{a}-\mathrm{pH}}+1}\cdot \left[{\mathrm{AcSCh}}^{+}\right]≅k_{\mathrm{oxime}}\cdot \frac{1}{10^{pK_{a}-\mathrm{pH}}+1}\cdot c\left({\mathrm{AcSCh}}^{+}\right)-k_{0}\cdot \;\frac{1}{10^{pK_{a}-\mathrm{pH}}+1}$$

where *k*_0_ represents possible competing pathway leading to oximate formation at the specified pH. The rates of oximolysis exceeded hydrolysis of AcSCh^+^ present in high molar excess such that the reaction 3 on Scheme 1 had a negligible contribution to the consumption of thioester in our experiments (see text below). The rates of consumption of the oximate correspond to the difference of the rates of oximolysis (reaction 1) and the neutral (H_2_O) and HO^−^-induced hydrolysis of the produced pyridinium-4-oxime-ester, AcOxime^+^, (reaction 2) which can be expressed as:

(4) $$k_{0}={\left(k_{w}+k_{\mathrm{OH}}\cdot \left[{\mathrm{HO}}^{-}\right]\right)}_{{\mathrm{AcOxime}}^{+}}$$

**Figure 1:**
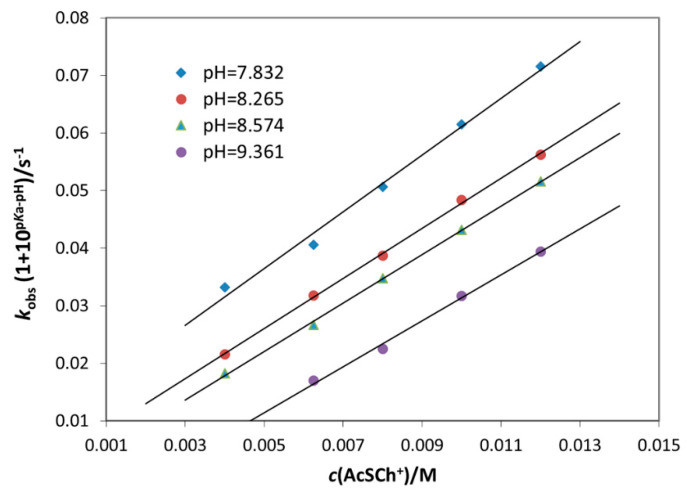
Observed rate constants for oximolysis of AcSCh^+^ by BPA4 at different pH of the medium, *I* = 0.1 M and 25 °C.

The second-order rate constant, *k*_oxime_, was determined from the slopes of the linear plots in Figure 1 and Figure S1 at 25 °C and *I* = 0.1 M and was found to be pH-independent in accordance with Equation (3).

**Scheme 2:**
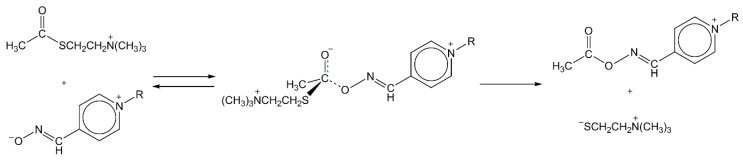
Mechanism of AcSCh^+^ oximolysis.

The matching values of *k*_oxime_ for both pyridinium-4-oximes, 3.81 M^−1^∙s^−1^ for PAM4 and 3.97 M^−1^∙s^−1^ for BPA4 (*I* = 0.1 M, 25 °C), are found to be in accordance with the mechanistic insight provided by the DFT computational studies. The computational analysis of reaction 1 points to the most common stepwise mechanism (Scheme 2) [21] and the formation of tetrahedral intermediate as the rate-limiting step with the $\Delta G_{\mathrm{COMP}}^{‡}$ of 68.6 kJ∙mol^−1^ for PAM4 and 69.0 kJ∙mol^−1^ for BPA4.

Thermodynamic activation parameters $\Delta H_{\mathrm{EXP}}^{‡}$ (kJ∙mol^−1^), $\Delta S_{\mathrm{EXP}}^{‡}$ (J∙K^−1^·mol^−1^) and $\Delta G_{\mathrm{EXP}}^{‡}$ (kJ∙mol^−1^) for the AcSCh^+^ oximolysis by PAM4 were determined over the temperature range 25–37 °C in BR buffer at pH = 8.3 and *I* = 0.1 M (Figure S1). The evaluated value of 69.45 kJ∙mol^−1^ for $\Delta G_{\mathrm{EXP}}^{‡}$, along with negative $\Delta S_{\mathrm{EXP}}^{‡}$ supports the theoretical mechanistic study.

The above described acetylation reaction stage (oximolysis) indeed mimics only the first stage of the catalytic process of the serine hydrolases, such as AcChE, but the complete enzyme catalyzed process includes two successive stages, acetylation and deacetylation, that is, hydrolysis of seryl-acetyl-ester formed in active site of the AcChE [25]. Assuming that oximate promotes the hydrolysis of AcSCh^+^ acting as pseudo-hydrolase/thioesterase, further investigation of the fate of pyridinium-4-oxime-ester (acetylated oxime) was necessary to provide mechanistic features of the second, deacetylation, stage of the total catalytic process. For that purpose, *O*-acetyl-*N*-methylpyridinium-4-oxime iodide (AcPAM4^+^I^−^) was synthesized and the independent kinetic study of the AcPAM4^+^ hydrolysis was performed (reaction 2 on Scheme 1). The progress of AcPAM4^+^ overall hydrolysis was followed spectrophotometrically at 25 °C and *I* = 0.1 M over the pH range from 6 to 12 where the rate of hydrolysis is dominated by neutral (H_2_O) and HO^−^ specific components, explicitly:

(5) $$k_{\mathrm{obs},\mathrm{hydrolysis}}=k_{w}+k_{\mathrm{OH}}\cdot \left[{\mathrm{HO}}^{-}\right]$$

The lowest value of the *k*_obs_ obtained in pure water (*k*_obs,hydrolysis_ = 1.5 × 10^−5^ s^−1^) was taken as the value for *k*_w_ (*k*_w_ > *k*_OH_·[HO^−^] and hence *k*_obs,hydrolysis_ ≅ *k*_w_), see Figure S2. The kinetic barrier, that is, the free energy of activation $\Delta G_{\mathrm{EXP}}^{‡}$ (kJ∙mol^−1^) for the neutral hydrolysis was calculated according to transition state theory [26] as follows:

(6) $$\Delta G^{‡}=R\cdot T\cdot ln\frac{k_{B}\cdot T}{h\cdot k_{w}}$$

where *k*_B_ and *h* are Boltzmann’s and Planck’s constants, respectively, *R* is the gas constant.

**Scheme 3:**
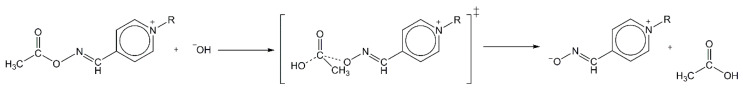
Mechanism of the AcOxime^+^ HO^−^-catalyzed hydrolysis.

Equation (6) gives $\Delta G_{\mathrm{EXP}}^{‡}$ = 100.5 kJ∙mol^−1^ at 25 °C and along with the value of *k*_w_ indicates insignificant contribution of the neutral AcPAM4^+^ hydrolysis to the observed oximolysis rate constant (see Equations (3) and (4)). Profile of the observed rates of AcPAM4^+^ hydrolysis vs. pH is presented in Figure S3a. The HO^−^-catalyzed hydrolysis appears to be the major hydrolytic mode above the pH 8.5 (since, *k*_OH_·[HO^−^] >> *k*_w,_ the *k*_obs,hydrolysis_ ≅ *k*_OH_·[HO^−^]). Therefore, the second-order rate constant, *k*_OH_, value of 30.3 M^−1^∙s^−1^ was evaluated, in the pH range from 9 to 11, as the slope of the straight line resulting from plot of the observed rate of hydrolysis (*k*_obs,hydrolysis_/s^−1^) against the concentration of HO^−^ (Figure S3b). The computational analysis of HO^−^-catalyzed AcPAM4^+^ hydrolysis points to the concerted mechanism and the formation of tetrahedral transition state with the kinetic barrier ($\Delta G_{\mathrm{COMP}}^{‡}$) of 35.1 kJ∙mol^−1^. It is reasonable to assume that the concerted mechanism is valid for hydrolysis of both pyridinium-4-oxime-esters and in the alkaline media can be presented as in Scheme 3.

The competitive reactions to AcSCh^+^ oximolysis by pyridinium-4-oximes are neutral and HO^−^-induced hydrolysis of AcSCh^+^ (overall hydrolysis, that is, reaction 3 on Scheme 1). To determine the influence of hydrolysis as a deleterious side reaction, the separate kinetic experiments were performed. The reactions were followed spectrophotometrically using the Ellman’s reagent [15] within the pH range from 7 to 9 at 25 °C and *I* = 0.1 M. We were forced to use the mild alkaline conditions due to the instability of Ellman’s reagent above pH 9 (see experimental for details). The rate constants *k*_w_ = 2.5 × 10^−5^ s^−1^ and *k*_OH_ = 59.0 M^−1^∙s^−1^ for hydrolysis of AcSCh^+^ were evaluated analogously as was described for hydrolysis of AcPAM4^+^, by using Equation (5). Profile of the observed rates of hydrolysis vs. pH and the data for the HO^−^-catalyzed hydrolysis are plotted in Figure S4. Again, the HO^−^-catalyzed hydrolysis appears to be the major hydrolytic mode above the pH 8.5 (Figure S4b). The *k*_w_ value for the neutral AcSCh^+^ hydrolysis agrees reasonable with the value reported for the neutral hydrolysis of formylthiocholine (*k*_w_ = 2.25 × 10^−5^ s^−1^ at 25 °C and *I* = 0.2 M) [20]. The mechanistic study also agrees with the stepwise reaction mechanism suggested for neutral formylthiocholine hydrolysis [20,21] and Equation (6) gives the kinetic barrier, $\Delta G_{\mathrm{EXP}}^{‡}$ = 99.3 kJ∙mol^−1^. The computational analysis of HO^–^-assisted AcSCh^+^ hydrolysis suggested the concerted mechanism with the kinetic barrier ($\Delta G_{\mathrm{COMP}}^{‡}$) of 26.8 kJ∙mol^−1^.

**Figure 2:**
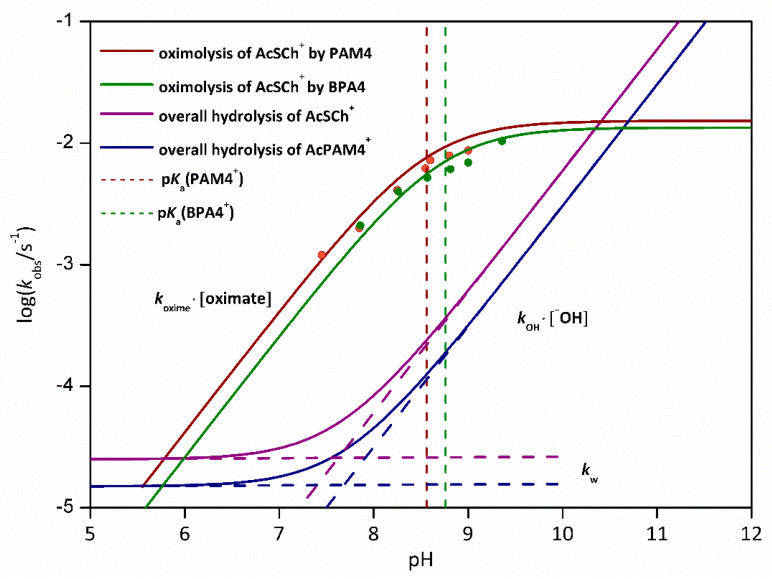
The pH-profiles of the total observed pseudo-first-order rate constants for the oximolysis of the AcSCh^+^ by PAM4 and BPA4 (reaction 1; *c*(AcSCh^+^) = 4 mM) and the neutral and HO^−^-induced hydrolysis of the AcSCh^+^ (reaction 3) and AcPAM4^+^ (reaction 2), respectively, at 25 °C.

The experimentally determined kinetic parameters are summarized in Table 1. Based on the values of kinetic constants, the pH-rate profiles are constructed and presented in Figure 2, illustrating several key features of the reaction systems.

**Figure 3:**
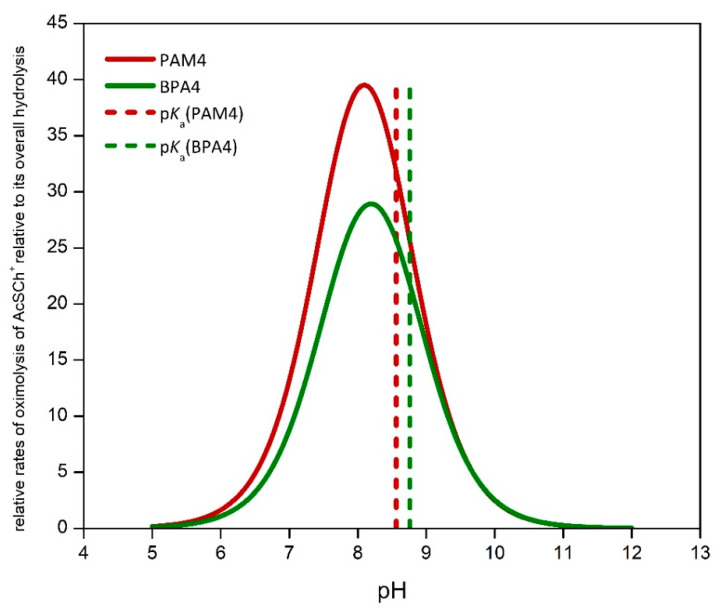
Plots of the calculated ratio of the observed rates of AcSCh^+^ oximolysis and aqueous overall hydrolysis as a function of pH. The relative rates were calculated as the ratio of Equations (3) and (5). The total concentration of AcSCh^+^ was 4 mM.

The shapes of the plots, that is, the rates of oximolysis are similar for both pyridinium-4-oximes suggesting that a phenyl substituent, distant from the reaction center, exerts practically no influence on the reaction outcomes. The rates of oximolysis scale with the concentrations of oximate and that is why rates plateau when pH reaches values of ~2 pH units higher than p*K*_a_(oxime). The rates of neutral hydrolysis of AcSCh^+^ and AcPAM4^+^ are constant, while the rates of HO^−^-induced hydrolysis scale with the concentrations of HO^−^. The HO^−^-catalyzed hydrolysis appears to be a predominant hydrolytic mode for both, AcSCh^+^ and AcPAM4^+^, above the pH of 8.5 (Figure 2, Figures S3 and S4). The minimum observed rates are in the pH regions below 6.5 of the pH-rate profiles and account for neutral hydrolysis of AcSCh^+^ and AcPAM4^+^. The pH-profile of the observed pseudo-first-order oximolysis rate constants relative to the overall (neutral and HO^−^-induced) observed rate of AcSCh^+^ hydrolysis is presented in Figure 3. The rates of AcSCh^+^ oximolysis by PAM4 or BPA4 are maximized relative to AcSCh^+^ overall hydrolysis at pH value of 8.1 and 8.2, respectively, where the oximolysis by PAM4 is ~40 times and by BPA4 ~ 30 times faster. A neutral and mildly basic aqueous media would have favored oximolysis with more acidic pyridinium-4-oximes providing the higher concentration of reactive nucleophile. Oximolysis by PAM4^+^ with the p*K*_a_ of 8.6, regardless the smaller value of *k*_oxime_ (*k*_oxime_ = 3.81 M^−1^∙s^−1^), is more favored than oximolysis by BPA4^+^ with the p*K*_a_ of 8.8 and *k*_oxime_ = 3.97 M^−1^∙s^−1^.

When considering the potential pseudo-hydrolase action of explored pyridinium-4-oximes, the corresponding catalytic cycle should be composed of two stages where, in the first acetylation one, (herein reaction 1) the thioester bond of AcSCh^+^ is oximolytically cleaved, while in the second deacetylation one (herein reaction 2) the ester bond of formed AcOxime^+^ is cleaved hydrolytically. In addition, to ensure the free progress of the catalytic cycles and appropriate regeneration of the oximate, the rates of reaction 1 and 2 must be comparable. Since the neutral AcOxime^+^ hydrolysis is very slow in comparison to its HO^−^-catalyzed hydrolysis and in regard to reaction 1, it can be assumed that the oximolysis and the formation of AcOxime^+^ is predominately followed by the HO^–^-catalyzed hydrolysis of AcOxime^+^ and restoration of the corresponding oximate, ending the catalytic cycle according to reactions 1 and 2 presented on Scheme 1. Accordingly, at the optimal pH value of 8.1 for the oximolysis by the PAM4 over the AcSCh^+^ overall hydrolysis (maximum of the curve in Figure 3, *k*_obs,oximolysis_ = 3.9 × 10^−3^ s^−1^, Equation (3)) the consecutive observed hydrolysis of AcPAM4^+^ proceeds almost 75-fold slower (*k*_obs,AcPAM4_^+^_hydrolysis_ = 5.3 × 10^−5^ s^−1^), recovering 1.36% of the oximate $(\frac{k_{{\mathrm{obs},\mathrm{AcPAM}4}^{+}\;\mathrm{hydrolysis}}}{k_{\mathrm{obs},\mathrm{hydrolysis}}}\cdot 100)$. This is further validated by the spectrophotometric study of the time-dependent electronic absorption spectral changes within reaction system containing PAM4 and 200-fold excess of AcSCh^+^ at pH around 8, *I* = 0.1 M and 25 °C (Figure S5).

**Figure 4:**
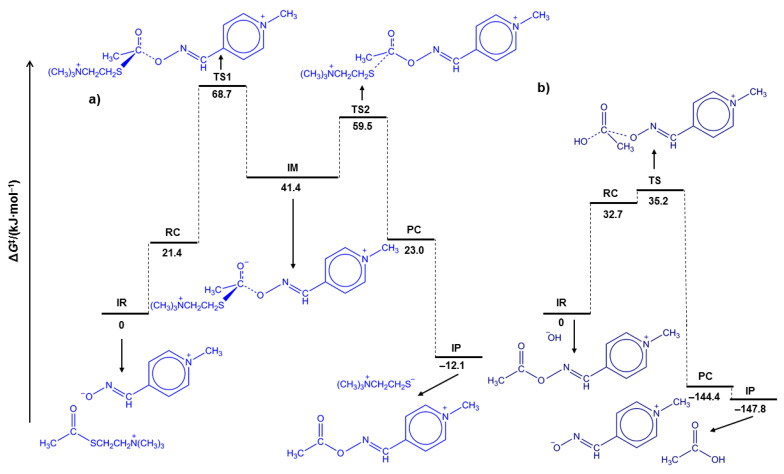
Free energy profiles for the (**a**) oximolysis of AcSCh^+^ by PAM4 and (**b**) HO^−^-mediated hydrolysis of AcPAM4^+^ (IR, isolated reactants; RC, reactant complex; TS1, first transition state; IM, intermediate; TS2, second transition state; PC, product complex; IP, isolated products). The reference point for all reactions is the energy of the isolated reactants.

The computational mechanistic study was performed using the (CPCM)/M06–2X/6–311++G(2df,2pd)//(CPCM)/M06–2X/6–31+G(d) model and the mechanistic data are summarized in Table 2 and Figure 4. The key geometric parameters for the reactant complex (RC), transition state (TS), intermediate (IM) and product complex (PC) of the acetylation and deacetylation stages of AcSCh^+^ hydrolysis are listed in Table S1.

First, let us observe that computational results indicate that the total Gibbs free energy of the reactant complex in reactions 1 and 2 is higher than the sum of the isolated reactants (Table 2). Nevertheless, these were found to be rather small, being in the range up to 33 kJ∙mol^−1^ and contribute to the calculated activation free energies. Yet, even then, the calculated $\Delta G_{\mathrm{COMP}}^{‡}$ values do not exceed 70 kJ∙mol^−1^ allowing these reactions to proceed under normal conditions. The latter is also facilitated by the fact that all reactions reported in Table 2 have negative reaction Gibbs energies and are favored thermodynamically, with the HO^−^-mediated AcSCh^+^ and AcPAM4^+^ hydrolysis being highly exergonic. Analogous to the mechanism of the AChE-catalyzed process, the oxime-mediated AcSCh^+^ hydrolysis consists of two successive stages—first, the oximolysis, that is, the acetylation stage characterized by the stepwise mechanism (Figure 4a) and second, the deacetylation stage, characterized by the concerted HO^−^-mediated AcOxime^+^ hydrolysis (Figure 4b). This is in accordance with the stepwise and concerted mechanisms found for acyl-transfer reactions of esters having weakly basic leaving groups [27]. Based on the calculated activation free Gibbs energies for the acetylation stage, the formation of TS1 by nucleophilic attack of an oximate to the carbonyl carbon atom of AcSCh^+^ represents the rate-determining step characterized with a kinetic parameter *k*_oxime_. The close resemblance between Δ*G*^‡^ values, calculated ($\Delta G_{\mathrm{COMP}}^{‡}$= 68.7 kJ∙mol^−1^) and experimentally determined ($\Delta G_{\mathrm{EXP}}^{‡}$ = 69.5 kJ∙mol^−1^) for the AcSCh^+^ oximolysis by PAM4, strongly support these computations. The changes of bond lengths and bond angles during the oximolysis (Figure 4a and Table S1) point to the formation of quasi-tetrahedral transition states (TS1 and TS2) and the intermediate (IM) in which the partial double character of the carbonyl bond is continuously preserved. Interestingly, the structure of the IM might suggest an intramolecular charge-charge stabilization, which would allow to expect the energy of this stationary point to be significantly lower than 41.1 kJ∙mol^−1^ above isolated reactants (IR) presented in Figure 4. However, our calculations show that the charged cationic amino group and the anionic carboxylate are located distant from each other during the course of the reaction, likely because of both (i) the resonance stabilization within the carboxylate, which dislocates anionic charge over several atoms and (ii) steric crowding surrounding the quaternary cationic amine, which hinders the exposure of the positive charge. As a result, there is not intramolecular interaction between these two fragments, additionally disfavored by the presence of the highly dielectric aqueous environment, thus the high-energy lying structure of the IM. Moreover, the structures of the first transition state (TS1) and IM indicate that the formation of the C_carbonyl_-O_oxime_ bond causes the substantial elongation of C-S rather than the carbonyl bond. Although the formation of the fully resolved tetrahedral intermediate within the stepwise mechanism should be expected and has been recognized for the methoxide- and AcChE-catalyzed AcSCh^+^ hydrolysis [25,28], our result could be rationalized with the postulation that the course of oximolysis is predominantly controlled by the cation-cation repulsive forces between thiocholine leaving group and pyridinium part of zwitterionic pyridinium-4-oximate. In the case of the second—deacetylation—stage characterized by the concerted HO^−^-mediated AcOxime^+^ hydrolysis (Figure 4b), the transition state (TS) structurally has a close resemblance to the initially formed reactant complex (RC) with a small degree of cleavage of the C_carbonyl_-O_oxime_ bond caused by the formation of C_carbonyl_--O_hydroxide_ close contact. Furthermore, the theoretical study points to the substantial mechanistic differences between the PAM4- and AcChE-mediated AcSCh^+^ hydrolysis. In the AcChE-mediated AcSCh^+^ hydrolysis both stages, acetylation and deacetylation, indeed occur through the stepwise mechanism, where within rate-limiting deacetylation stage the H_2_O as a nucleophile attacks the carbonyl carbon of the acetylated seryl-residue within the enzyme active site [25]. On the contrary, the stepwise acetylation stage of the PAM4-mediated AcSCh^+^ hydrolysis is rate-limiting process followed by the concerted attack of HO^−^ to the carbonyl carbon of the AcPAM4^+^ in the second, deacetylation stage. The involvement of H_2_O as a nucleophile in the implied stepwise deacetylation stage is ruled out, due to the evaluated high energy barrier ($\Delta G_{\mathrm{EXP}}^{‡}$ = 100.5 kJ∙mol^−1^) for the neutral AcPAM4^+^ hydrolysis when compared with HO^−^-catalyzed reaction ($\Delta G_{\mathrm{COMP}}^{‡}$ = 35.2 kJ∙mol^−1^). Our results of kinetic and theoretical mechanistic studies clearly show that the acetylation stage of AcSCh^+^ hydrolysis by PAM4 or BPA4 represents the rate-limiting stage of the investigated, overall, process.

The potential mimetic enzymatic performance of pyridinium-4-oximes toward AcSCh^+^ hydrolysis was further validated by taking in account that the neutral and pyridinium-4-oxime-catalyzed hydrolysis of AcSCh^+^ presumably occur via the equivalent stepwise mechanism. The experimentally determined kinetic parameters *k*_oxime_/M^−1^∙s^−1^ and $k_{w}$/s^−1^ were used for the calculation of the rate enhancement expressed as *k*_oxime_/$k_{w}^{\prime }$, where $k_{w}^{\prime }$ = 4.5 × 10^−7^ M^−1^·s^−1^, accounts for the second-order rate constant of neutral AcSCh^+^ hydrolysis ($k_{w}^{\prime }$ = $k_{w}$/55.56 M). The 8.5 × 10^6^- and 8.8 × 10^6^-fold rate increase of the PAM4- and BPA4-catalyzed AcSCh^+^ hydrolysis, respectively, puts the pyridinium-4-oximes into the category of very efficient and potent esterolytic agents. While important, this rate enhancement is modest in comparison to the 10^13^-fold catalytic power manifested by cholinesterases [16,17].

The presented results of AcSCh^+^ oximolysis by PAM4 and BPA4 reflect their equal nucleophilicity and insignificant effect of the benzene ring within BPA4. Moreover, the reaction with both oximes is exergonic (Δ*G*_R COMP_ = −12.1 kJ·mol^−1^ for PAM4 and Δ*G*_R COMP_ = −10.0 kJ∙mol^−1^ for BPA4), the difference being only 2.1 kJ∙mol^−1^ in favor of PAM4. The similar reactivity, that is, electronic properties of these two pyridinium-4-oximes has been recently established by the analysis of the corresponding frontier orbitals where it becomes clear that no significant electron density is observed in the additional phenyl ring in the LUMO of BPA4^+^, while the majority of the electron density is located in the pyridinium-4-oxime moiety, just as in PAM4^+^ [14]. As was previously recognized for the reactivity of pyridinium-oximes toward the AcSCh^+^, the reactivity differences did not origin from different electron density on the oxygen of the oximate but was caused with the steric hindrance of the oxime group by the rest of the molecule, resulting in higher reactivity of the oxime group in para-position than those in ortho-position [8,9,22]. It should be noted, that even though the greater acidity of oxime functional group ensures higher concentration of nucleophilic active oximate form in neutral solutions, that is, at physiological pH, the significant contribution on the nucleophilicity and consequent esterolytic activity has the steric effect, whether through structural characteristic of electrophilic center or nucleophile itself.

## 3. Materials and Methods

### 3.1. Chemicals and General Physical Measurements

The protocols for the synthesis of *N*-methylpyridinium-4-oxime iodide (PAM4^+^I^−^) and *N*-benzylpyridinium-4-oxime chloride monohydrate (BPA4^+^Cl^−^·H_2_O), their spectroscopic characterization (FTIR, FT-Raman, FT-MS, ^1^H- and ^13^C-NMR) and crystal structure parameters have been provided in previous studies [24,26]. All other reagents such as pyridine-4-oxime, acetylthiocholine iodide (Fluka, Charlotte, NC, USA), Elman’s reagent (5,5′-dithiobis-2-nitrobenzoic acid; DTNB) (Sigma-Aldrich, St. Louis, MO, USA), methyl iodide (ACROS-Organics, Waltham, MA, USA) and acetylchloride (Fluka) were reagent-grade chemicals and were used as purchased. The ultra-pure water (ASTM Type 1 quality) obtained using a Millipore Direct-Q5 purification system (MERCK, Burlington, MA, USA) was used to prepare the reaction mixtures. The Britton−Robinson (BR) buffers were prepared by combining a mixture of 0.04 M solutions of phosphoric, boric and acetic acid, respectively (20 mL) with appropriate volumes of 0.20 M NaOH solution. The tris(hydroxymethyl)aminomethane (THAM) buffer systems were prepared by the addition of different volumes of 0.50 M HCl solution to the aqueous solution of THAM with the concentration of acidic buffer component being held constant at 0.10 M.

The UV/Vis kinetic measurements were performed on a Varian Cary Bio 100 spectrophotometer (Agilent Technologies, Palo Alto, CA, USA) with thermostated cell holders using 1 cm cuvettes. The ^1^H and ^13^C NMR spectra were recorded at room temperature with a Bruker AV-600 spectrometer (Bruker, Billerica, MA, USA), operating at 600.13 and 150.9 MHz, respectively. FTIR spectrum was recorded on a Perkin Elmer Spectrum GX (PerkinElmer, Waltham, MA, USA), Series R spectrometer in the range of 4000–400 cm^−1^ using KBr pellets. Elemental analysis was performed by using a Perkin Elmer 2400 Series II CHNS elemental analyzer with accuracy percent of ±0.3%.

The single mass analysis was obtained with a ThermoFischer Scientific Orbitrap Exploris high resolution mass spectrometer (ThermoFischer, Waltham, MA, USA) using electrospray ionization. A Mettler Toledo pH-meter (Mettler Toledo, Columbus, OH, USA) with an open junction combination polymer electrode was used for pH measurements accurate to ±0.01 pH units.

#### Synthesis of *O*-Acetyl-*N*-methylpyridinium-4-oxime iodide (AcPAM4^+^I^−^)

The general synthetic route for the oxime acetylation [29,30] was adjusted and optimized to the pyridinium-4-oxime-ester synthesis. To the acetone solution of pyridine-4-oxime (700.0 mg; 5.7 mmol in 35 mL), three-fold excesses of triethylamine (2.4 mL; 17.1 mmol) and acetylchloride (1.2 mL; 17.1 mmol) were added. The reaction mixture was stirred at ambient temperature until the bright yellow precipitate containing predominantly triethylamonium chloride was quantitatively formed. The solid was removed by filtration and the iodomethane (0.70 mL; 11.4 mmol) was added to the remaining clear acetone solution. The reaction mixture was stirred for 2 h and then left overnight in the dark at ambient temperature allowing the precipitation of the yellow AcPAM4^+^I^−^ powder. The final product was filtered off, washed several times with acetone and diethyl ether and then dried in the desiccator under reduced pressure over P_4_O_10_. The mass of the solid was 895.1 mg (η = 51.5%). Anal. (%): Calcd. for C_9_H_11_N_2_O_2_I: C, 35.32; H, 3.62; N, 9.15. Found: C, 35.43; H, 3.34; N, 9.06.

ESI–MS *m*/*z*: Calcd. for C_9_H_11_N_2_O_2_ [M − I]^+^: 179.0821. Found 179.0902. UV-spectral analysis in water at 25 °C: *λ*_max_ = 255 nm, *ε* = 20456 M^−1^∙cm^−1^ (π→π* CT band). FT-IR (cm^−1^): *ν*(C=O)_ester_, 1781 (vs); *ν*(N-O-C=O)_oxime-ester_, 1707 (w); *ν*(C=N)_oxime_, 1644 (vs); *ν*(C-C, C-N)_pyridinium ring_, 1610 (w), 1567 (s), 1524 (s); *ν*(C-O)_ester_, 1198 (vs); *ν*(N-O)_oxime_, 1004 (vs). ^1^H NMR (600.133 MHz, DMSO-*d*_6_, 25 °C, TMS): δ (ppm): 9.10 (d, *J* = 6.48 Hz, 2H; PyH), 8.97 (s, 1H, CH=N), 8.39 (d, *J* = 6.48 Hz, 2H; PyH), 4.39 (s, 3H; CH_3_-N^+^_py_), 2.28 (s, 3H; CH_3_-C=O). ^13^C NMR (150.197 MHz, DMSO-*d*_6_, 25 °C, TMS): δ (ppm): 167.8 (Ac, C=O), 152.7 (1C; PyC), 146.4 (CH=N), 145.2 (2C; PyC), 125.5 (2C; PyC), 48.2 (C-N^+^_py_), 19.3 (Ac, CH_3_).

Note: the AcPAM4^+^I^−^ should be stored in the airtight bottle, at cool and dry place, due to hydrolytic instability when exposed to humid air.

### 3.2. Kinetic Studies

A standard kinetic experiment was performed by injecting freshly prepared stock solutions of reactant(s) into a thermally equilibrated solution contained in a cuvette at constant ionic strength of 0.1 M, adjusted when necessary, by the addition of NaCl. The spectrophotometric monitoring of the PAM4 or BPA4 disappearance was performed at absorption maximum (337 nm or 340 nm, respectively [24,26]). The observed pseudo-first order rate constants of oximolysis, *k*_obs,oximolysis_ (s^−1^) and HO^−^-mediated AcPAM4^+^ hydrolysis, *k*_obs,hydrolysis_ (s^−1^), were determined from nonlinear curve fits of absorbance versus time plots for at least 90% of the reaction progress with correlation coefficients >0.99. The pseudo-first order rate constants of AcSCh^+^ hydrolysis and neutral AcPAM4^+^ hydrolysis, *k*_obs,hydrolysis_ (s^−1^), were determined as slopes from the linear plots of ln(*A*_max_ − *A*_t_) versus time at 412 nm or ln(*A*_t_) versus time at 300 nm, respectively. All kinetic measurements were performed at least in triplicate and the values of the kinetic parameters, *k*_oxime_ (M^−1^∙s^−1^), *k*_w_ (s^‒1^) and *k*_OH_ (M^−1^∙s^−1^) were given as mean values with experimental error less than 5%.

#### 3.2.1. The Kinetic Details of Oximolysis Reaction

In each kinetic experiment performed at constant BR-buffered pH value (range from 7.9 to 9.1), the initial concentration of corresponding pyridinium-4-oxime, *c*(oxime), was held constant and equal to 4.0 × 10^−5^ M while the excess concentration of AcSCh^+^, *c*(AcSCh^+^), was varied from 4.0 × 10^−3^ to 15.5 × 10^−3^ M. The high excess of AcSCh^+^ assured large observed rate values of oximolysis at specified pH and insignificant contribution of the H_2_PO_4_^−^ buffer component to the hydrolysis of AcSCh^+^. The thermodynamic activation parameters, Δ*H*^‡^ and Δ*S*^‡^ for the AcSCh^+^ oximolysis by PAM4 were determined from the Eyring linear plots of ln(*k*_oxime_/*T*) versus 1/*T,* using the determined *k*_oxime_ (M^−1^∙s^−1^) values of 3.81, 5.67 and 9.08 at 25.1 °C, 30.2 °C and 37.3 °C, respectively (inset in Figure S1). The equation Δ*G*^‡^ = Δ*H*^‡^ − *T*∙Δ*S*^‡^ was used to calculate the corresponding $\Delta G_{\mathrm{EXP}}^{‡}$ value at 25 °C.

#### 3.2.2. The Kinetic Details of AcPAM4^+^ Hydrolysis

The rates of AcPAM4^+^ hydrolysis were followed spectrophotometrically under pseudo-first-order conditions in pure water (*c*(AcPAM4^+^) = 4.5 × 10^−5^ M; pH = 7.0), BR-buffered solutions (*c*(AcPAM4^+^) = 1.3 × 10^−5^ M; pH = 6.0–11.2) and in the presence of NaOH (*c*(AcPAM4^+^) = 1.3 × 10^‒5^ M; pH= 10.1–11.2) at 25 °C and *I* = 0.1 M. The pseudo-first-order rate constant *k*_obs, hydrolysis_/s^−1^, identified as *k*_w_, was determined by spectrophotometric monitoring of the time-dependent change in electronic absorption spectra of AcPAM4^+^ at 300 nm (the isosbestic point of PAM4^+^/PAM4 system absorbing species) in pure water and in BR-buffered solution of pH = 6.0 (Figure S2).

#### 3.2.3. The Kinetic Details of AcSCh^+^ Hydrolysis

The rates of AcSCh^+^ hydrolysis were determined spectrophotometrically by using the Ellman’s reagent DTNB and recording the time-depended change in electronic absorption spectra of reaction mixtures (*c*(AcSCh^+^) = 5 × 10^−5^ M; *c*(DTNB) = 2.5 × 10^−5^ M) at 25 °C and *I* = 0.1 M. The Ellman’s reagent is not stable in highly alkaline solutions due to the reaction with HO^−^ ions [31,32]. Therefore, the rates were studied within the narrow pH range from 7.0 to 9.0. The pH was kept constant by using THAM-buffer system which is chosen because of its relatively poor nucleophilic properties [17].

### 3.3. Computational Details

All molecular geometries were optimized and thermal corrections to Gibbs free energies extracted at the efficient M06–2X/6–31+G(d) model without the application of the scaling factors, which are, for this level of theory, proposed to be very close to 1 [33] and would not exhibit a significant effect on the obtained vibrational frequencies. The final single-point energies were attained with a highly flexible 6–311++G(2df,2pd) basis set using the M06–2X functional, which was designed to provide highly accurate thermodynamic and kinetic parameters for various organic systems. To account for the effects of the aqueous solution we included, during both the geometry optimization and the single-point energy evaluation, a conductor-like polarizable continuum model (CPCM) with all of the parameters for pure water, giving rise to the (CPCM)/M06–2X/6–311++G(2df,2pd)//(CPCM)/M06–2X/6–31+G(d) model employed here, being in line with our earlier reports on similar systems [11,14,34,35]. Additionally, no basis set superposition error correction was employed as both the size and the flexibility of the utilized 6–311++G(2df,2pd) basis set warrants that such corrections would only be marginal. All transition state structures were located using the scan procedure, employing both 1D and 2D scans, the latter specifically utilized to exclude the possibility for concerted mechanisms. Apart from the visualization of the obtained negative frequencies, the validity of all transition state structures was validated by performing IRC calculations in both directions and identifying the matching reactant and product structures connected by the inspected transition state. In the reaction profiles, the energy of the isolated reactants (IRs) and analogously for the isolated products (IPs), correspond to the sum of total Gibbs free energies of the individual reacting partners and defines the reference point for the other calculated stationary points. All of the calculations were performed using the Gaussian 16 software [36].

## 4. Conclusions

In conclusion, the question which still remains is can the pyridinium-4-oxime be classified as a non-enzyme catalyst toward the AcSCh^+^ hydrolysis? To answer it, as precisely as possible, the following facts should be taken into account. The complete pyridinium-4-oximate pseudo-hydrolase catalytic cycle consists of two stages—first one is the oximolytic cleavage of AcSCh^+^ (reaction 1 on Scheme 1 which represents acetylation stage) and second one which represents consequent hydrolytic cleavage of formed AcOxime^+^ (reaction 2 on Scheme 1 or deacetylation stage) that leads to the regeneration of active pyridinium-4-oximate catalyst and the closure of catalytic cycle. In addition, the rate enhancement of AcSCh^+^ oximolysis over AcSCh^+^ hydrolysis should be considered along with the rates comparison of oximolysis of AcSCh^+^ and hydrolysis of AcOxime^+^. The optimal pH-value for the oximolytic cleavage of AcSCh^+^ established for examined pyridinium-4-oximes is an average of 8.15 and at this pH the side-reaction to reaction 1 is the overall hydrolysis of AcSCh^+^ (reaction 3 on Scheme 1). The observed rate enhancement greater than 10^6^ on behalf of AcSCh^+^ oximolysis over the neutral AcSCh^+^ hydrolysis reveals the very large potency of pyridinium-4-oximes in the first stage of catalytic cycle. Also, at this pH, the fate of the formed AcOxime^+^ is determined by its overall hydrolysis. By comparing the observed rate constants of stage 1 and stage 2 at above mentioned pH it turns out that the rate of oximolysis is almost one hundred times greater than those of hydrolysis stage. Given the rate at which the second stage proceeds, we can assume that it is insufficient, so the pyridinium-4-oxime cannot be classified as non-enzyme catalyst of the AcSCh^+^ hydrolysis but as the very effective oximolytic/esterolytic agent.

## Figures and Tables

**Table 1:** Kinetic and activation parameters at 25 °C and *I* = 0.1 M.

**Oximolysis Reaction**				
**Ester**	**Nucleophile**	***k*_oxime_ (M^−1^∙s^−1^)**	$\Delta G_{EXP}^{‡}$ **(kJ∙mol^−1^)**	$\Delta S_{EXP}^{‡}$ **(J∙K^−1^∙mol^−1^)**
AcSCh^+^	PAM4	3.81 ± 0.21	69.9 ± 3.2	−56.1 ± 2.6
	BPA4	3.97 ± 0.25	NM	NM
**Neutral and HO^−^-Catalyzed Hydrolysis of Esters**				
**Ester**	**Nucleophile**	***k*_w_ (s^−1^)**	***k*_OH_ (M^−1^∙s^−1^)**	$\Delta G_{EXP}^{‡}$ **(kJ∙mol^−1^)**
AcSCh^+^	OH_2_	(2.5 ± 0.2) × 10^−5^		99.3 ± 4.5
	HO^−^		59.0 ± 3.1	
AcPAM4^+^	OH_2_	(1.5 ± 0.3) × 10^−5^		100.5 ± 5.2
	HO^−^		30.3 ± 1.3	

**Table 2:** Computed mechanisms, kinetic (RC, reactant complex; TS, transition state; IM, intermediate; PC, product complex) and thermodynamic (Δ*G*_R COMP_) parameters for the specified reactions at 25 °C, with energies in kJ∙mol^−1^. The reference point for all reactions is the energy of the isolated reactants.

Reaction ^a^	Nucleophile	Mechanism	RC	TS_1_	IM	TS_2_	PC	Δ*G*^‡^	Δ*G*_R_^b^
1	PAM4	stepwise	21.4	68.7	41.4	59.5	23.0	68.7	−12.1
1	BPA4	stepwise	18.0	69.1	48.1	63.6	25.1	69.1	−10.0
2 ^c^	HO^−^	concerted	32.7	35.2	-	-	−144.4	35.2	−147.8
3	HO^−^	concerted	−5.0	26.8	-	-	−116.0	31.8	−159.9

^a^ Numeration was made according to Scheme 1. ^b^ The energy of the initial state (isolated reactants, IR) was set to 0 kJ∙mol^−1^ and Δ*G*_R_ = IP − IR; (isolated products, IP). ^c^ The reaction 2 refers to HO^−^-catalyzed hydrolysis of AcPAM4^+^.

## Supplementary Materials

The following are available online, Figure S1: Observed rate constants for the oximolysis of AcSCh^+^ by PAM4 at different temperatures, *I* = 0.1 M and pH = 8.3, Figure S2: The neutral AcPAM4^+^ hydrolysis (by H_2_O) at 25 °C and *I* = 0.1 M presented by time-dependent change of AcPAM4^+^ electronic absorption spectrum, Figure S3: (**a**) Profile of the observed rates of AcPAM4^+^ hydrolysis vs. pH; (**b**) A plot of the observed rate of hydrolysis (*k*_obs_) vs. *c*(HO^–^), adjusted with addition of 0.20 M NaOH solution for the specific OH^−^-mediated hydrolysis of AcPAM4^+^ at 25 °C and *I* = 0.1 M, Figure S4: (**a**) Profile of the observed rates of hydrolysis vs. pH for AcSCh^+^; (**b**) A plot of the observed rate of hydrolysis (*k*_obs_) vs. the concentration of hydroxide ion, *c*(HO^–^), for the HO^−^-mediated hydrolysis of AcSCh^+^ at 25 °C and *I* = 0.1 M, Figure S5: Time-dependent changes in the UV–Vis spectra of the PAM4 solution in the presence of 200-fold excess of AcSCh^+^ at 25 °C and *I* = 0.1 M, pH = 7.9, Table S1: List of key geometric parameters for the reactant complex (RC), transition state (TS), intermediate (IM) and product complex (PC) of the acetylation and deacetylation stages of the AcSCh^+^ hydrolysis obtained by the (CPCM)/M06–2X/6–31+G(d) computational model, Cartesian coordinates, total molecular energies, thermal corrections to Gibbs free energies of all computationally investigated systems.
