# Supplementary material for: Novel Insights into the Thioesterolytic Activity of N-Substituted Pyridinium-4-oximes
Source: Molecules. 2020 May 21;25(10):2385. doi: 10.3390/molecules25102385 (PMC7287890; doi:10.3390/molecules25102385)
Supplement: Supplementary file 1 [file molecules-25-02385-s001.pdf]

Novel insights into the thioesterolytic activity of *N*-substituted pyridinium-4-oximes

Blaženka Foretić [1,*], Vladimir Damjanović [1], Robert Vianello [2] and Igor Picek [1,*]

1 Department of Chemistry and Biochemistry, School of Medicine, University of Zagreb, Šalata 3, HR-10000 Zagreb, Croatia; bforetic@mef.hr (B.F.); vladimir.damjanovic@mef.hr (V.D.); ipicek@mef.hr (I.P.)
2 Division of Organic Chemistry and Biochemistry, Ruđer Bošković Institute, Bijenička 54, HR-10000 Zagreb, Croatia; Robert.Vianello@irb.hr (R.V.)
* Correspondence: bforetic@mef.hr (B.F.); ipicek@mef.hr (I.P.)

Contents:

Figure S1: Observed rate constants for the oximolysis of $AcSCh^+$ by PAM4 at different temperatures, I = 0.1 M and pH = 8.3.

Figure S2: The neutral $AcPAM4^+$ hydrolysis (by H_2O) at 25 °C and I = 0.1 M presented by time-dependent change of $AcPAM4^+$ electronic absorption spectrum.

Figure S3: a) Profile of the observed rates of $AcPAM4^+$ hydrolysis *vs*. pH.; b) A plot of the observed rate of hydrolysis (k_{obs}) *vs*. $c(HO^-)$, adjusted with addition of 0.20 M NaOH solution for the specific OH^--mediated hydrolysis of $AcPAM4^+$ at 25 °C and I = 0.1 M.

Figure S4: a) Profile of the observed rates of hydrolysis *vs*. pH for $AcSCh^+$; b) A plot of the observed rate of hydrolysis (k_{obs}) *vs.* the concentration of hydroxide ion, $c(HO^-)$, for the HO^--mediated hydrolysis of $AcSCh^+$ at 25 °C and I = 0.1 M.

Figure S5: Time-dependent changes in the UV–Vis spectra of the PAM4 solution in the presence of 200-fold excess of $AcSCh^+$ at 25 °C and I = 0.1 M, pH = 7.9.

Table S1: List of key geometric parameters for the reactant complex (RC), transition state (TS), intermediate (IM) and product complex (PC) of the acetylation and deacetylation stages of the $AcSCh^+$ hydrolysis obtained by the (CPCM)/M06–2X/6–31+G(d) computational model.

Cartesian coordinates, total molecular energies, thermal corrections to Gibbs free energies and geometries of all computationally investigated systems.

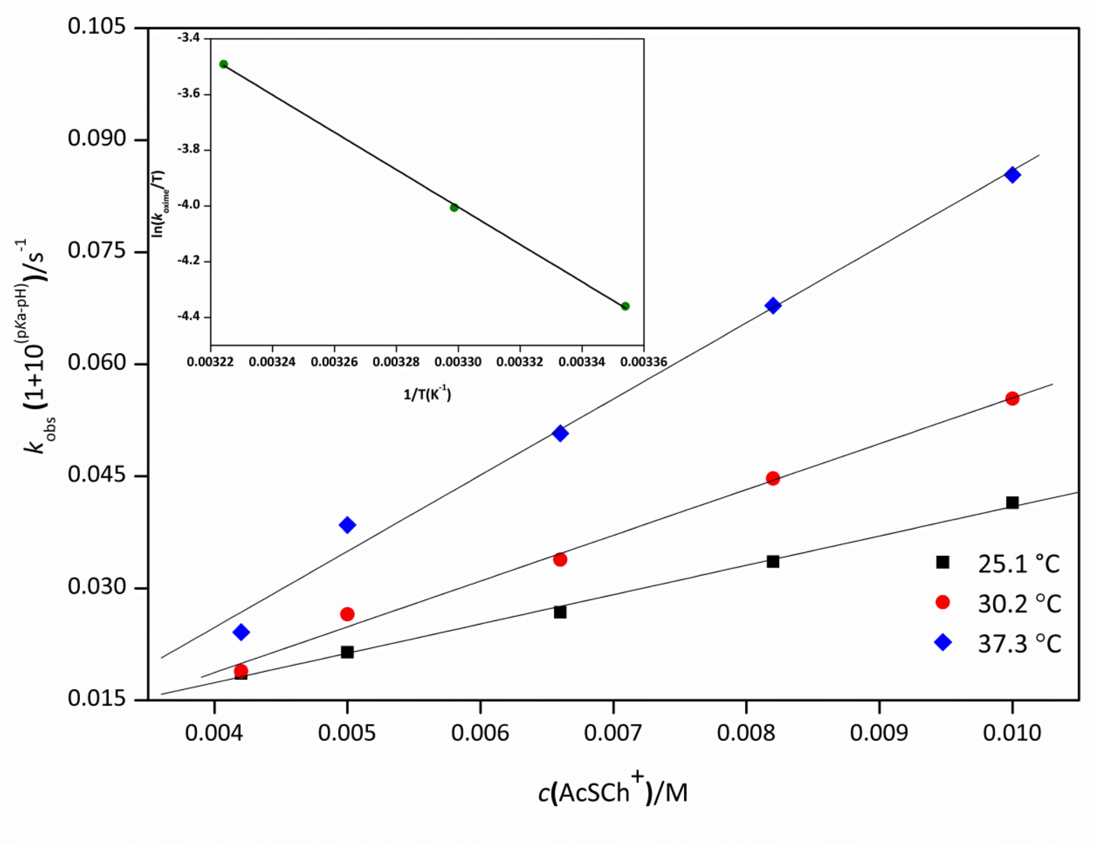

Figure S1. Observed rate constants for the oximolysis of $AcSCh^+$ by PAM4 at different temperatures, I = 0.1 M and pH = 8.3. Inset represents Eyring linear plot. The $\Delta G^{‡}$ value was calculated at 25 °C by using the equation: $\Delta G^{‡} = \Delta H^{‡} - T \cdot \Delta S^{‡}$.

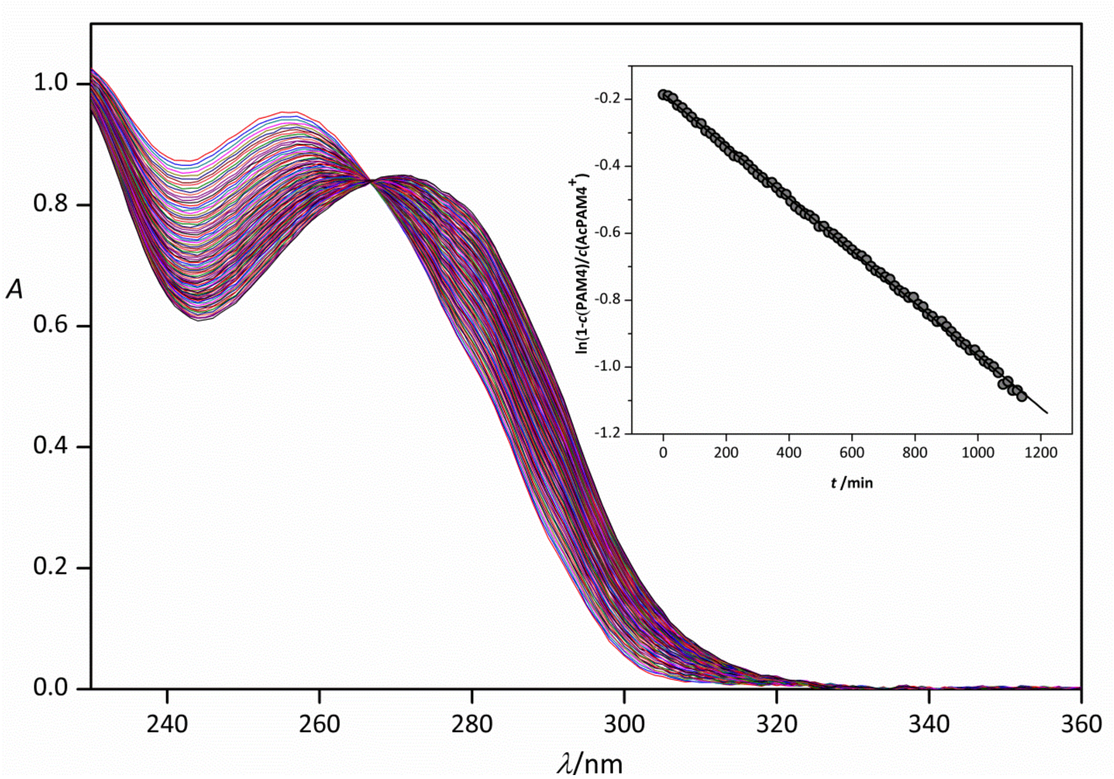

Figure S2. The neutral $AcPAM4^+$ hydrolysis (by H_2O) at 25 °C and I = 0.1 M presented by time-dependent change of $AcPAM4^+$ electronic absorption spectrum. The pseudo-first-order rate constant, k_W (s^{-1}), was determined as k_{obs} using the *isosbestic point method* according to the: $\ln(1-c(PAM4)/c(AcPAM4^+)) = k_{obs}\cdot t$ (see inset).

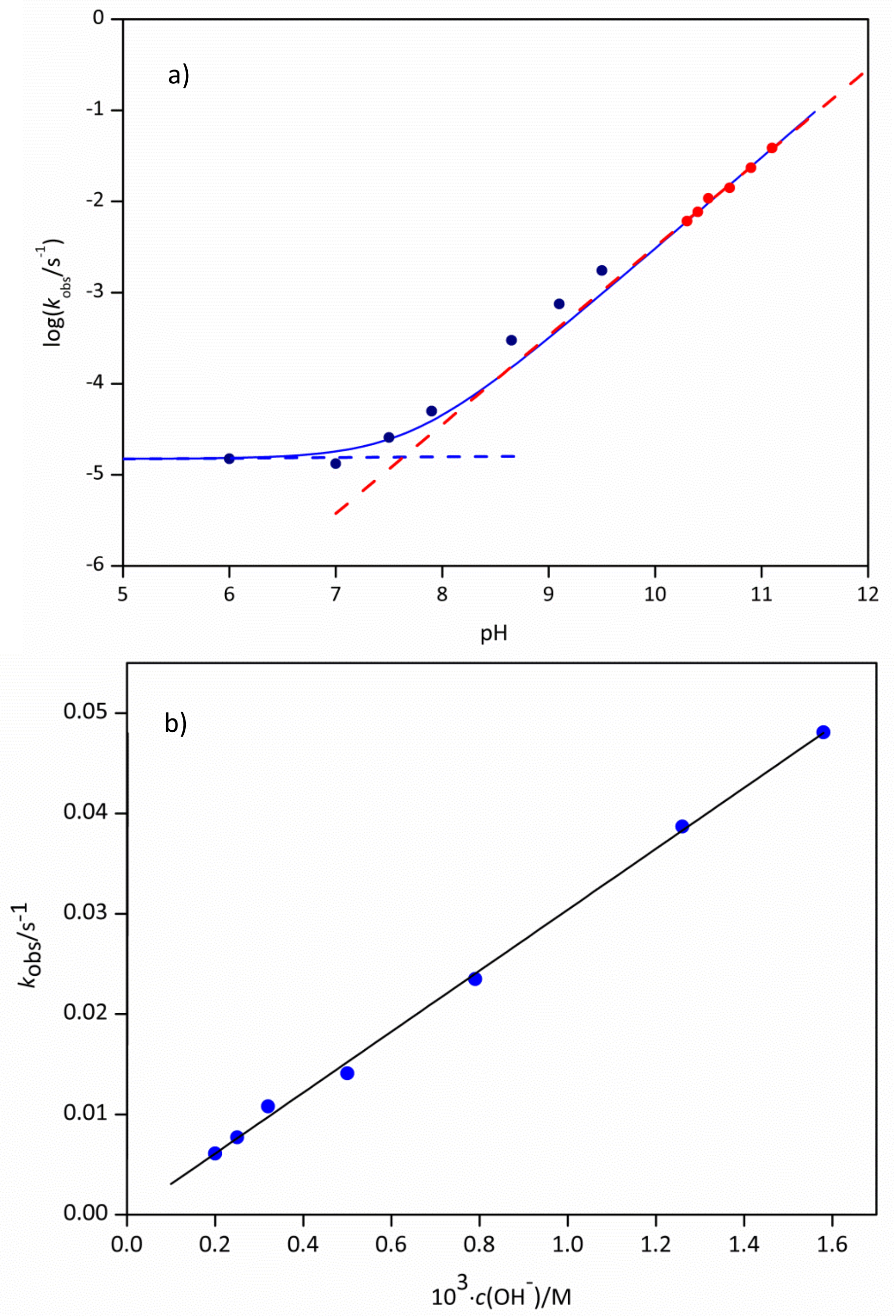

Figure S3. a) Profile of the observed rates of AcPAM4$^+$ hydrolysis *vs*. pH. The slanting dashed line (red) on the log-log plot have slope of 1 and represents the region where HO^--catalyzed hydrolysis predominates. b) A plot of the observed rate of hydrolysis (k_{obs}) *vs*. $c(HO^-)$, adjusted with addition of 0.20 M NaOH solution for the specific OH^--mediated hydrolysis of AcPAM4$^+$ at 25 °C and I = 0.1 M. The slope of the regression line is k_{OH} = 30.345 $M^{-1}\cdot s^{-1}$, with $r^2 > 0.99$.

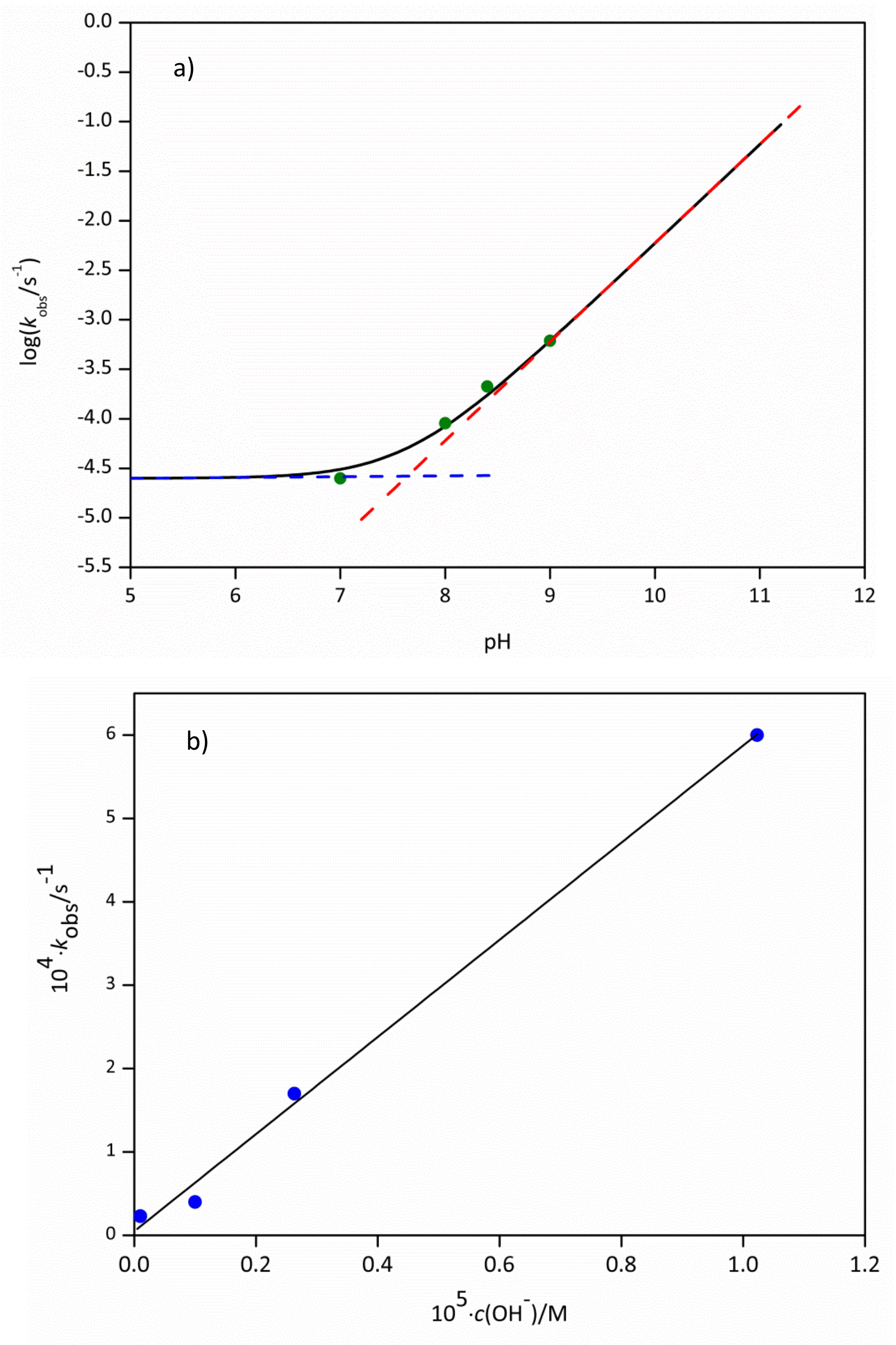

Figure S4. a) Profile of the observed rates of hydrolysis *vs*. pH for $AcSCh^+$. The slanting dashed line (red) on the log-log plot have slope of 1 and represents the region where HO^--catalyzed hydrolysis predominates. b) A plot of the observed rate of hydrolysis (k_{obs}) *vs.* the concentration of hydroxide ion, $c(HO^-)$, for the HO^--mediated hydrolysis of $AcSCh^+$ at 25 °C and I = 0.1 M. The slope of the regression line is k_{OH} = 59.006 $M^{-1} \cdot s^{-1}$, with $r^2 > 0.99$.

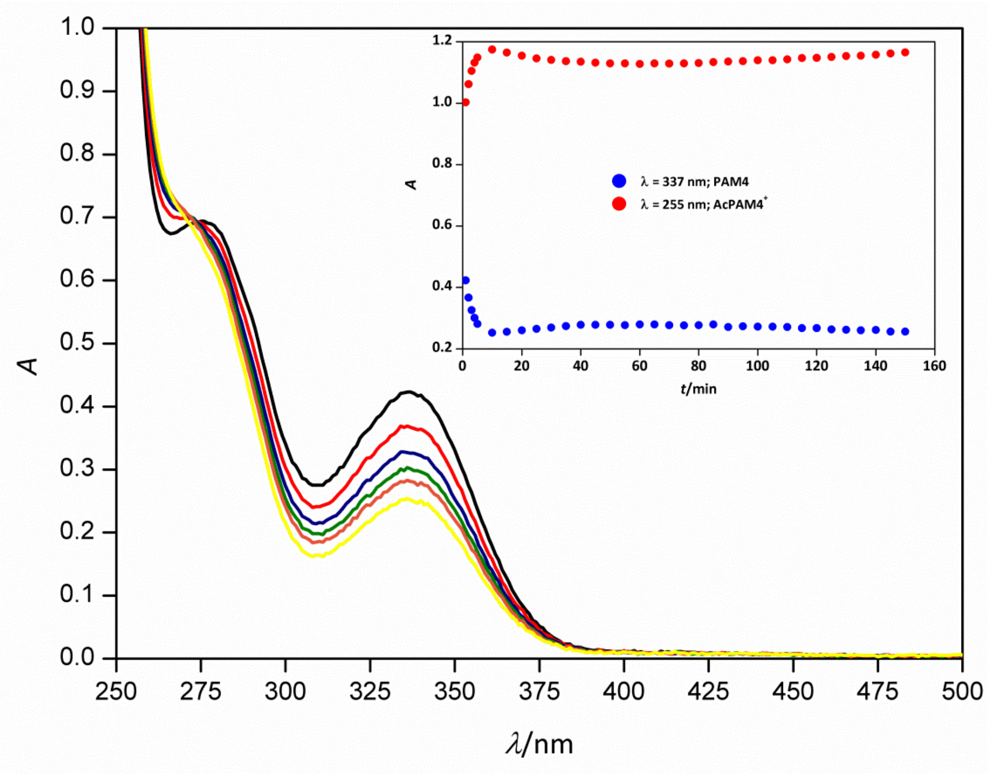

Figure S5. Time-dependent changes in the UV–Vis spectra of the PAM4 solution in the presence of 200-fold excess of $AcSCh^+$ at 25 °C and I = 0.1 M, pH = 7.9 (BR buffer). Inset: the mirror-like time-dependent change in absorbance at 337 nm and 255 nm reflects change in concentrations of PAM4 and $AcPAM4^+$, respectively, during the course of $AcSCh^+$ hydrolysis, showing that oximolysis and the formation of $AcOxime^+$ is followed by the HO^--catalyzed hydrolysis of $AcOxime^+$ and restoration of the PAM4.

Table S1. List of key geometric parameters for the reactant complex (RC), transition state (TS), intermediate (IM) and product complex (PC) of the acetylation and deacetylation stages of the $AcSCh^{+}$ hydrolysis obtained by the (CPCM)/M06–2X/6–31+G(d) computational model.

	$AcSCh^{+}$ oximolysis by PAM4 (acetylation stage of $AcSCh^{+}$ hydrolysis)					
	bond lengths (Å)			bond angles (°)		
	$C_{carbonyl}$–O_{oxime}	$(C–O)_{carbonyl}$	$(C–S)_{AcSCh+}$	∠(O_{oxime}–C=O)	∠(H_3C–C=O)	∠(S–C=O)
RC	3.011	1.209	1.799	83.2	124.4	121.4
TS1	1.934	1.228	1.877	107.9	123.0	117.4
IM	1.507	1.257	2.055	115.3	119.8	111.9
TS2	1.456	1.236	2.279	118.0	122.4	108.0
PC	1.374	1.203	3.456	123.3	126.6	94.5
	$AcPAM4^{+}$ hydrolysis by OH^{-} (deacetylation stage of $AcSCh^{+}$ hydrolysis)					
	bond lengths (Å)			bond angles (°)		
	$C_{carbonyl}$–O_{oxime}	$(C–O)_{carbonyl}$	$C_{carbonyl}$–$O_{hydroxide}$	∠(O_{oxime}–C=O)	∠(H_3C–C=O)	∠(O=C–$O_{hydroxide}$)
RC	1.389	1.203	2.551	122.5	127.3	101.1
TS1	1.396	1.205	2.407	122.0	127.0	102.0
PC	4.038	1.220	1.328	85.4	124.3	122.9
	$AcSCh^{+}$ oximolysis by BPA4 (acetylation stage of $AcSCh^{+}$ hydrolysis)					
	bond lengths (Å)			bond angles (°)		
	$C_{carbonyl}$–O_{oxime}	$(C–O)_{carbonyl}$	$(C–S)_{AcSCh+}$	∠(O_{oxime}–C=O)	∠(H_3C–C=O)	∠(S–C=O)
RC	2.979	1.209	1.799	83.9	124.4	121.5
TS1	1.929	1.228	1.878	108.1	123.0	117.4
IM	1.508	1.257	2.055	115.1	119.9	111.9
TS2	1.457	1.236	2.271	118.1	122.3	108.3
PC	1.374	1.203	3.448	123.2	126.6	93.6

Cartesian coordinates, total molecular energies, thermal corrections to Gibbs free energies of all computationally investigated systems

Structure:	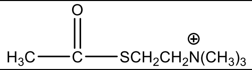
Geometry:	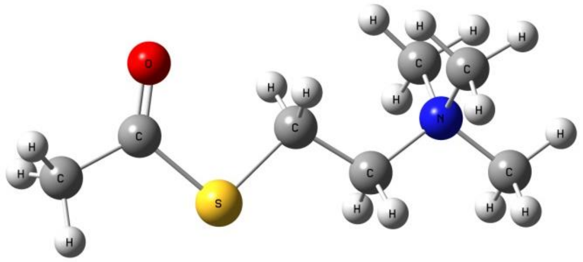
System:	**isolated reactant (IR):** reaction 1, nucleophile PAM4
(CPCM)/M06–2X/6–311++G(2df,2pd) Electronic Energy (in a.u.):	-804.33933
(CPCM)/M06–2X/6–31+G(d) Thermal Correction to Gibbs Free Energy (in a.u.)	0.193617
Number of imaginary frequencies:	0

CARTESIAN COORDINATES

Center Number	Atomic Number	Atomic Type	Coordinates (Angstroms) X	Y	Z
1	6	0	4.261853	-0.229831	0.048585
2	6	0	2.860363	0.318751	-0.013506
3	8	0	2.592764	1.497983	-0.024038
4	16	0	1.586887	-0.935930	-0.030073
5	6	0	0.135952	0.164624	-0.012409
6	1	0	4.599350	-0.181273	1.089094
7	1	0	4.315600	-1.265963	-0.290077
8	1	0	4.914965	0.400578	-0.558253
9	6	0	-1.098850	-0.725601	-0.013187
10	1	0	0.198464	0.787186	0.881949
11	1	0	0.187694	0.801426	-0.897233
12	7	0	-2.406609	0.034831	0.007108
13	1	0	-1.125988	-1.351459	-0.908780
14	1	0	-1.111996	-1.374324	0.866177
15	6	0	-2.526682	0.868152	1.246624
16	6	0	-2.537932	0.912652	-1.200030
17	6	0	-3.516655	-0.974692	-0.005495
18	1	0	-3.527686	1.298086	1.270497
19	1	0	-2.368479	0.228460	2.115259
20	1	0	-1.786956	1.666248	1.218298
21	1	0	-4.467911	-0.443188	0.011746
22	1	0	-3.434862	-1.572045	-0.913309
23	1	0	-3.422463	-1.607935	0.876508
24	1	0	-3.539855	1.341237	-1.201026
25	1	0	-1.799178	1.710620	-1.148142
26	1	0	-2.385026	0.305206	-2.092499

Structure:	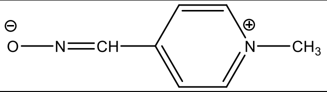
Geometry:	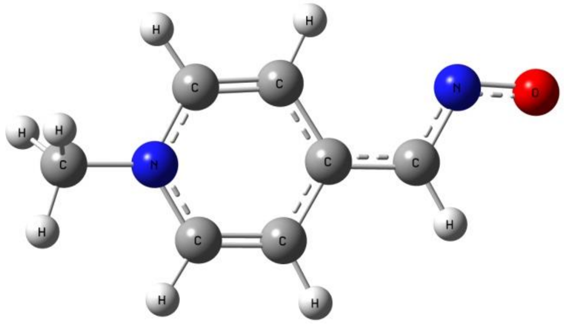
System:	**isolated reactant (IR):** reaction 1, nucleophile PAM4
(CPCM)/M06–2X/6–311++G(2df,2pd) Electronic Energy (in a.u.):	-456.16824
(CPCM)/M06–2X/6–31+G(d) Thermal Correction to Gibbs Free Energy (in a.u.)	0.109491
Number of imaginary frequencies:	0

CARTESIAN COORDINATES

Center Number	Atomic Number	Atomic Type	Coordinates (Angstroms) X	Y	Z
1	6	0	0.046939	-1.007066	-0.000304
2	6	0	0.624561	0.295273	-0.000119
3	6	0	-0.292565	1.379133	-0.000150
4	6	0	-1.644456	1.156869	-0.000220
5	7	0	-2.154522	-0.097485	-0.000364
6	6	0	-1.310226	-1.163494	-0.000394
7	6	0	2.024211	0.532743	0.000054
8	7	0	2.864719	-0.495218	0.000122
9	8	0	4.104757	-0.223817	0.000317
10	1	0	2.412236	1.552994	0.000157
11	1	0	0.066839	2.402310	-0.000143
12	1	0	-2.365751	1.965402	-0.000211
13	1	0	-1.784847	-2.138369	-0.000503
14	1	0	0.680545	-1.885017	-0.000412
15	6	0	-3.605060	-0.328892	0.000722
16	1	0	-4.113299	0.633479	-0.004414
17	1	0	-3.882643	-0.893955	-0.890063
18	1	0	-3.882942	-0.884774	0.897216

Structure:	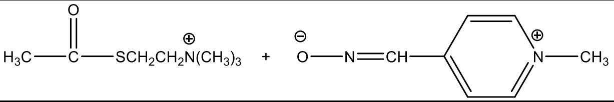
Geometry:	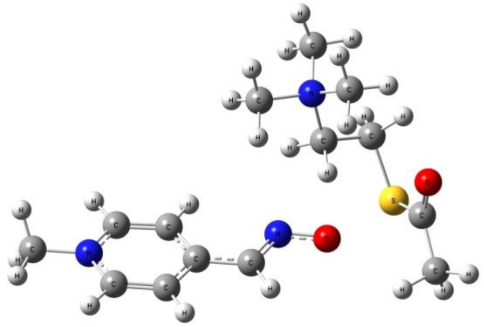
System:	**reactant complex (RC):** reaction 1, nucleophile PAM4
(CPCM)/M06–2X/6–311++G(2df,2pd) Electronic Energy (in a.u.):	-1260.52122
(CPCM)/M06–2X/6–31+G(d) Thermal Correction to Gibbs Free Energy (in a.u.)	0.324813
Number of imaginary frequencies:	0

CARTESIAN COORDINATES

Center Number	Atomic Number	Atomic Type	Coordinates (Angstroms) X	Y	Z
1	6	0	-3.394225	-3.169519	0.037488
2	6	0	-3.564647	-1.679272	-0.039199
3	8	0	-3.663947	-1.055213	-1.069870
4	16	0	-3.574424	-0.855830	1.560007
5	6	0	-3.414325	0.846322	0.948050
6	1	0	-3.760927	-3.577269	0.981849
7	1	0	-3.904927	-3.638140	-0.805877
8	1	0	-2.318458	-3.364366	-0.040561
9	6	0	-1.961917	1.061730	0.528132
10	1	0	-4.119367	0.958160	0.121995
11	1	0	-3.715469	1.507213	1.762793
12	7	0	-1.756341	2.194011	-0.454871
13	1	0	-1.348780	1.293772	1.402777
14	1	0	-1.559149	0.168778	0.040259
15	6	0	-2.266935	1.793718	-1.804706
16	6	0	-2.435415	3.443758	0.003013
17	6	0	-0.282124	2.441245	-0.559898
18	1	0	-2.080942	2.613134	-2.499381
19	1	0	-1.735080	0.892932	-2.113670
20	1	0	-3.333861	1.587255	-1.742469
21	1	0	-0.108283	3.183761	-1.339047
22	1	0	0.076869	2.811317	0.401079
23	1	0	0.204449	1.495533	-0.806130

24	1	0	-2.169935	4.251597	-0.679238
25	1	0	-3.514203	3.290585	-0.008017
26	1	0	-2.094279	3.675764	1.012762
27	8	0	-0.623824	-1.571924	-0.674074
28	7	0	0.459695	-1.006384	-0.311722
29	6	0	1.595370	-1.551045	-0.712404
30	6	0	2.829946	-0.952807	-0.326064
31	6	0	4.062824	-1.485809	-0.777221
32	6	0	5.252829	-0.913196	-0.407096
33	7	0	5.292071	0.172886	0.398704
34	6	0	4.131575	0.712236	0.855235
35	6	0	2.914556	0.186237	0.521324
36	1	0	4.082346	-2.355564	-1.424523
37	1	0	6.209827	-1.298040	-0.738490
38	6	0	6.570640	0.786174	0.786729
39	1	0	4.235892	1.578251	1.498995
40	1	0	2.015446	0.641216	0.918105
41	1	0	1.585615	-2.446108	-1.336780
42	1	0	6.616324	1.803713	0.396409
43	1	0	6.648814	0.798482	1.874422
44	1	0	7.384139	0.196178	0.369100

Structure:	
Geometry:	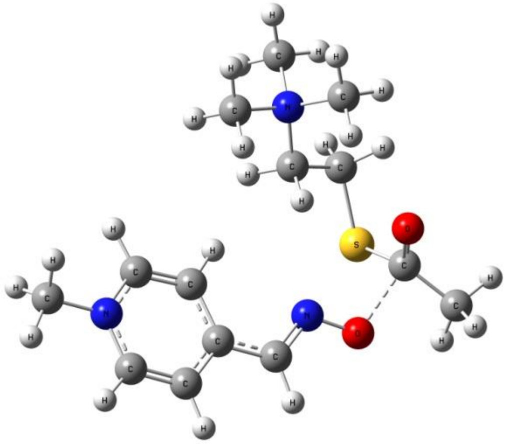
System:	**transition state 1 (TS1)**: reaction 1, nucleophile PAM4
(CPCM)/M06–2X/6–311++G(2df,2pd) Electronic Energy (in a.u.):	-1260.50714
(CPCM)/M06–2X/6–31+G(d) Thermal Correction to Gibbs Free Energy (in a.u.)	0.328818
Number of imaginary frequencies:	1 (–200 cm^{-1})

CARTESIAN COORDINATES

Center Number	Atomic Number	Atomic Type	Coordinates (Angstroms) X	Y	Z
1	6	0	3.271230	-3.105262	0.473657
2	6	0	2.574062	-1.767465	0.365879
3	8	0	2.510922	-0.969153	1.296850
4	16	0	2.711055	-1.062185	-1.367670
5	6	0	2.911798	0.680002	-0.901057
6	1	0	3.060550	-3.739470	-0.390085
7	1	0	4.349157	-2.923504	0.528679
8	1	0	2.942220	-3.606161	1.387402
9	6	0	1.566859	1.273353	-0.503000
10	1	0	3.631521	0.715961	-0.081712
11	1	0	3.335817	1.186539	-1.770952
12	7	0	1.667061	2.578910	0.263543
13	1	0	0.968299	1.482619	-1.394517
14	1	0	1.029244	0.577455	0.144806
15	6	0	2.282893	2.350740	1.610818
16	6	0	2.470834	3.585023	-0.496486
17	6	0	0.280809	3.107646	0.462550
18	1	0	2.236218	3.286344	2.168510
19	1	0	1.721349	1.566174	2.117362
20	1	0	3.319696	2.043974	1.489429
21	1	0	0.340800	4.051592	1.004445

22	1	0	-0.181460	3.263830	-0.512577
23	1	0	-0.286673	2.380904	1.044958
24	1	0	2.451915	4.527578	0.051236
25	1	0	3.496884	3.229718	-0.583158
26	1	0	2.027503	3.712366	-1.484892
27	8	0	0.844873	-2.617152	0.195198
28	7	0	-0.090371	-1.732464	0.111784
29	6	0	-1.310101	-2.186863	0.170695
30	6	0	-2.420186	-1.272757	0.062012
31	6	0	-3.739622	-1.733939	0.234081
32	6	0	-4.799706	-0.862279	0.137678
33	7	0	-4.605799	0.447034	-0.125736
34	6	0	-3.350087	0.924081	-0.308762
35	6	0	-2.258020	0.100663	-0.226259
36	1	0	-3.939555	-2.777562	0.449764
37	1	0	-5.827166	-1.178888	0.268956
38	6	0	-5.745659	1.374667	-0.226907
39	1	0	-3.271089	1.983230	-0.526484
40	1	0	-1.269983	0.506953	-0.397449
41	1	0	-1.501620	-3.251933	0.308013
42	1	0	-5.632362	2.160565	0.520078
43	1	0	-5.767111	1.805821	-1.228059
44	1	0	-6.664259	0.821275	-0.043947

Structure:	
Geometry:	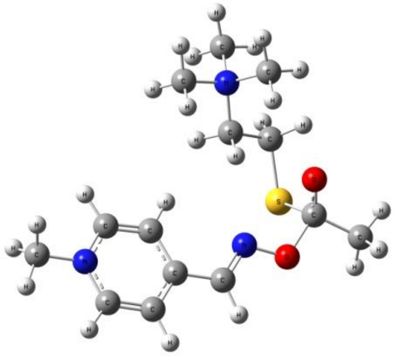
System:	**intermediate (IM)**: reaction 1, nucleophile PAM4
(CPCM)/M06–2X/6–311++G(2df,2pd) Electronic Energy (in a.u.):	-1260.51045
(CPCM)/M06–2X/6–31+G(d) Thermal Correction to Gibbs Free Energy (in a.u.)	0.329844
Number of imaginary frequencies:	0

CARTESIAN COORDINATES

Center Number	Atomic Number	Atomic Type	Coordinates (Angstroms) X	Y	Z
1	6	0	-2.882616	-3.301083	-0.485271
2	6	0	-2.066583	-2.016238	-0.421729
3	8	0	-2.219150	-1.135070	-1.304968
4	16	0	-2.405003	-1.274853	1.465225
5	6	0	-2.994223	0.354699	0.928426
6	1	0	-2.675447	-3.962928	0.359618
7	1	0	-3.942701	-3.038848	-0.484008
8	1	0	-2.643968	-3.820524	-1.420520
9	6	0	-1.802745	1.206877	0.517855
10	1	0	-3.670866	0.194189	0.086634
11	1	0	-3.547786	0.802031	1.757486
12	7	0	-2.164268	2.471662	-0.244147
13	1	0	-1.240116	1.530473	1.398564
14	1	0	-1.157054	0.628500	-0.146120
15	6	0	-2.755303	2.129146	-1.577403
16	6	0	-3.127460	3.306932	0.535132
17	6	0	-0.907666	3.251438	-0.468857
18	1	0	-2.882488	3.054091	-2.140981
19	1	0	-2.077057	1.446094	-2.088372
20	1	0	-3.720374	1.648276	-1.431128
21	1	0	-1.152471	4.162769	-1.014941
22	1	0	-0.468151	3.497578	0.498079
23	1	0	-0.220602	2.640088	-1.054459
24	1	0	-3.289381	4.242078	-0.001640
25	1	0	-4.069135	2.767937	0.629777
26	1	0	-2.702211	3.504918	1.519933

27	8	0	-0.663002	-2.545435	-0.277163
28	7	0	0.247652	-1.565513	-0.205505
29	6	0	1.449536	-2.008695	-0.187452
30	6	0	2.555553	-1.057251	-0.079871
31	6	0	3.875442	-1.508034	-0.191483
32	6	0	4.919597	-0.610093	-0.091899
33	7	0	4.686061	0.699076	0.114688
34	6	0	3.420573	1.163028	0.233403
35	6	0	2.344435	0.313484	0.144022
36	1	0	4.099210	-2.555576	-0.358914
37	1	0	5.957014	-0.909605	-0.174404
38	6	0	5.805985	1.654513	0.223311
39	1	0	3.317058	2.228160	0.404521
40	1	0	1.341846	0.705791	0.257006
41	1	0	1.669999	-3.074804	-0.254134
42	1	0	5.702728	2.407205	-0.557992
43	1	0	5.775191	2.121667	1.207496
44	1	0	6.740757	1.113224	0.097592

Structure:	
Geometry:	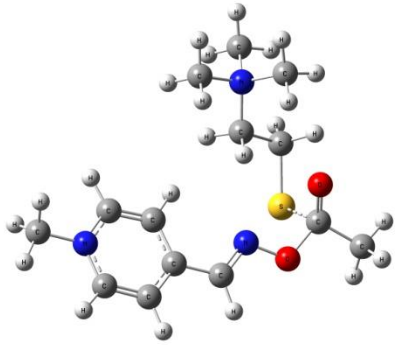
System:	**transition state 2 (TS2)**: reaction 1, nucleophile PAM4
(CPCM)/M06–2X/6–311++G(2df,2pd) Electronic Energy (in a.u.):	-1260.51026
(CPCM)/M06–2X/6–31+G(d) Thermal Correction to Gibbs Free Energy (in a.u.)	0.328488
Number of imaginary frequencies:	1 (–159 cm^{-1})

CARTESIAN COORDINATES

Center Number	Atomic Number	Atomic Type	Coordinates (Angstroms) X	Y	Z
1	6	0	-2.861094	-3.313954	-0.507418
2	6	0	-1.994297	-2.072130	-0.519256
3	8	0	-2.162135	-1.155470	-1.331410
4	16	0	-2.405924	-1.250373	1.566027
5	6	0	-3.028689	0.335637	0.931758
6	1	0	-2.644864	-3.952556	0.350594
7	1	0	-3.908160	-3.009162	-0.480666
8	1	0	-2.674480	-3.872082	-1.433294
9	6	0	-1.844105	1.202293	0.534039
10	1	0	-3.655362	0.120672	0.062353
11	1	0	-3.638263	0.815911	1.701267
12	7	0	-2.201170	2.454480	-0.255351
13	1	0	-1.303665	1.546492	1.420907
14	1	0	-1.172905	0.626012	-0.106245
15	6	0	-2.811444	2.088408	-1.573108
16	6	0	-3.145996	3.318355	0.514540
17	6	0	-0.938959	3.214584	-0.511478
18	1	0	-2.908613	2.997131	-2.168159
19	1	0	-2.161445	1.363769	-2.063715
20	1	0	-3.793166	1.649870	-1.405221
21	1	0	-1.181452	4.124585	-1.060974
22	1	0	-0.477068	3.463067	0.444457
23	1	0	-0.271836	2.588629	-1.104837
24	1	0	-3.312332	4.237902	-0.047435
25	1	0	-4.088480	2.787936	0.642319
26	1	0	-2.702340	3.543358	1.485258

27	8	0	-0.637844	-2.553715	-0.301487
28	7	0	0.270669	-1.556829	-0.254162
29	6	0	1.466550	-2.002096	-0.172655
30	6	0	2.570854	-1.043674	-0.070859
31	6	0	3.891670	-1.490899	-0.169163
32	6	0	4.931009	-0.585813	-0.073737
33	7	0	4.688340	0.723657	0.117061
34	6	0	3.420652	1.183157	0.223776
35	6	0	2.348956	0.326669	0.137401
36	1	0	4.121396	-2.539183	-0.323156
37	1	0	5.970474	-0.880469	-0.146893
38	6	0	5.802807	1.686545	0.222477
39	1	0	3.311177	2.249361	0.383903
40	1	0	1.343393	0.713959	0.243033
41	1	0	1.687025	-3.070155	-0.180134
42	1	0	5.702851	2.426759	-0.571009
43	1	0	5.758945	2.167694	1.199316
44	1	0	6.741418	1.148298	0.114327

Structure:	
Geometry:	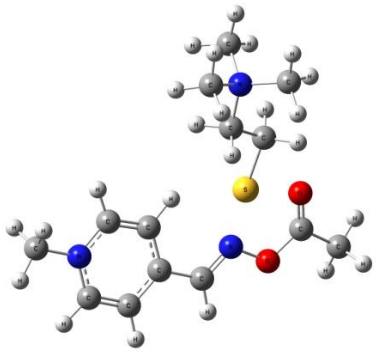
System:	**product complex (PC)**: reaction 1, nucleophile PAM4
(CPCM)/M06–2X/6–311++G(2df,2pd) Electronic Energy (in a.u.):	-1260.52160
(CPCM)/M06–2X/6–31+G(d) Thermal Correction to Gibbs Free Energy (in a.u.)	0.325964
Number of imaginary frequencies:	0

CARTESIAN COORDINATES

Center Number	Atomic Number	Atomic Type	Coordinates (Angstroms) X	Y	Z
1	6	0	-2.524965	-3.394949	-0.850916
2	6	0	-1.607793	-2.220343	-0.999819
3	8	0	-1.865097	-1.172436	-1.531569
4	16	0	-2.489070	-1.141600	2.163404
5	6	0	-3.246810	0.111192	1.057506
6	1	0	-2.261432	-3.994354	0.019955
7	1	0	-3.548695	-3.029162	-0.766282
8	1	0	-2.442649	-4.014715	-1.750190
9	6	0	-2.168066	1.091202	0.628753
10	1	0	-3.664156	-0.385264	0.176773
11	1	0	-4.061569	0.628368	1.574775
12	7	0	-2.637814	2.241955	-0.261323
13	1	0	-1.704684	1.564751	1.498549
14	1	0	-1.401442	0.564975	0.054976
15	6	0	-3.453867	1.733043	-1.406550
16	6	0	-3.440490	3.223880	0.528309
17	6	0	-1.425227	2.929475	-0.801138
18	1	0	-3.620642	2.556724	-2.101732
19	1	0	-2.905285	0.923048	-1.888035
20	1	0	-4.408129	1.369181	-1.027910
21	1	0	-1.739043	3.809979	-1.362719
22	1	0	-0.788165	3.221621	0.034290
23	1	0	-0.897300	2.234743	-1.454935

24	1	0	-3.757609	4.028792	-0.135846
25	1	0	-4.312113	2.720359	0.943705
26	1	0	-2.815371	3.620335	1.328886
27	8	0	-0.362075	-2.531794	-0.511968
28	7	0	0.531866	-1.481654	-0.550606
29	6	0	1.661318	-1.854638	-0.094230
30	6	0	2.757601	-0.874747	-0.028771
31	6	0	4.024120	-1.292981	0.381320
32	6	0	5.058293	-0.376838	0.440975
33	7	0	4.853929	0.909788	0.109542
34	6	0	3.635736	1.340902	-0.286249
35	6	0	2.570014	0.473163	-0.363220
36	1	0	4.220238	-2.324179	0.652293
37	1	0	6.060402	-0.645639	0.750642
38	6	0	5.959265	1.886478	0.196882
39	1	0	3.557405	2.393476	-0.531164
40	1	0	1.602358	0.841705	-0.681070
41	1	0	1.838838	-2.874857	0.246929
42	1	0	6.001438	2.449004	-0.734734
43	1	0	5.768615	2.554242	1.037171
44	1	0	6.890842	1.346641	0.349117

Structure:	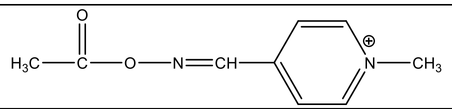
Geometry:	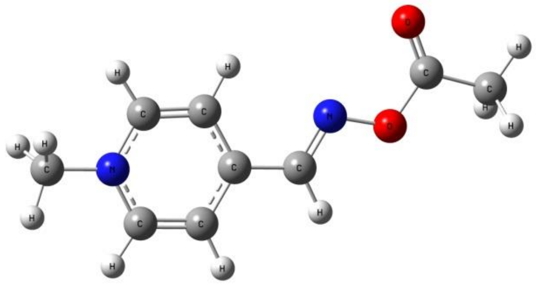
System:	**isolated product (IP)**: reaction 1, nucleophile PAM4
(CPCM)/M06–2X/6–311++G(2df,2pd) Electronic Energy (in a.u.):	-609.27984
(CPCM)/M06–2X/6–31+G(d) Thermal Correction to Gibbs Free Energy (in a.u.)	0.154211
Number of imaginary frequencies:	0

CARTESIAN COORDINATES

Center Number	Atomic Number	Atomic Type	Coordinates (Angstroms) X	Y	Z
1	6	0	1.196332	-0.864408	-0.057966
2	6	0	0.813359	0.483353	-0.027291
3	6	0	1.807029	1.461288	0.020258
4	6	0	3.136867	1.081512	0.042032
5	7	0	3.478888	-0.218243	0.015950
6	6	0	2.534847	-1.184430	-0.035198
7	6	0	-0.601381	0.891753	-0.038787
8	7	0	-1.495737	-0.014533	-0.013818
9	8	0	-2.753280	0.556796	-0.024992
10	6	0	-3.781731	-0.358145	0.020620
11	1	0	-0.849883	1.953208	-0.062372
12	1	0	1.562556	2.517064	0.044270
13	1	0	3.947548	1.798364	0.079701
14	1	0	2.895990	-2.205805	-0.057334
15	1	0	0.458356	-1.656448	-0.097682
16	6	0	4.902054	-0.617470	0.022045
17	1	0	5.511331	0.259845	0.225216
18	1	0	5.050512	-1.363515	0.801583
19	1	0	5.151730	-1.031993	-0.954724
20	8	0	-3.608435	-1.544549	0.067178
21	6	0	-5.090586	0.371796	0.001463
22	1	0	-5.140366	1.065651	0.844396
23	1	0	-5.173854	0.954172	-0.919888
24	1	0	-5.902988	-0.350577	0.061362

Structure:	$\overset{\ominus}{S}HCH_2CH_2\overset{\oplus}{N}H_3(CH_3)_3$
Geometry:	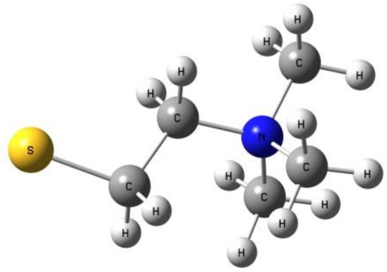
System:	**isolated product (IP)**: reaction 1, nucleophile PAM4
(CPCM)/M06–2X/6–311++G(2df,2pd) Electronic Energy (in a.u.):	-651.23314
(CPCM)/M06–2X/6–31+G(d) Thermal Correction to Gibbs Free Energy (in a.u.)	0.149700
Number of imaginary frequencies:	0

CARTESIAN COORDINATES

Center Number	Atomic Number	Atomic Type	Coordinates (Angstroms) X	Y	Z
1	16	0	2.893900	0.194179	-0.000032
2	6	0	1.231439	-0.587013	0.000121
3	6	0	0.180220	0.510361	-0.000464
4	1	0	1.120814	-1.220681	-0.884328
5	1	0	1.120636	-1.220004	0.885034
6	7	0	-1.274507	0.042496	-0.000027
7	1	0	0.286873	1.139257	0.887269
8	1	0	0.286739	1.138123	-0.889019
9	6	0	-1.571708	-0.768954	-1.219812
10	6	0	-1.571729	-0.766432	1.221416
11	6	0	-2.147269	1.257795	-0.001219
12	1	0	-2.638720	-0.993459	-1.235198
13	1	0	-1.295282	-0.188338	-2.100653
14	1	0	-1.000224	-1.694651	-1.181921
15	1	0	-3.190980	0.942714	-0.000437
16	1	0	-1.930747	1.843701	0.892156
17	1	0	-1.931384	1.841535	-0.896158
18	1	0	-2.638639	-0.991413	1.236914
19	1	0	-0.999775	-1.691917	1.185734
20	1	0	-1.295878	-0.183746	2.101063

Structure:	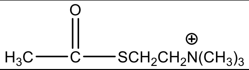
Geometry:	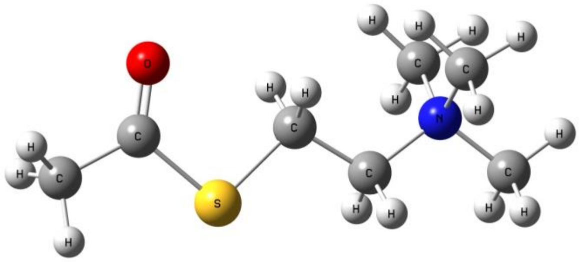
System:	**isolated reactant (IR):** reaction 1, nucleophile BPA4
(CPCM)/M06–2X/6–311++G(2df,2pd) Electronic Energy (in a.u.):	-804.33933
(CPCM)/M06–2X/6–31+G(d) Thermal Correction to Gibbs Free Energy (in a.u.)	0.193617
Number of imaginary frequencies:	0

CARTESIAN COORDINATES

Center Number	Atomic Number	Atomic Type	Coordinates (Angstroms) X	Y	Z
1	6	0	4.261853	-0.229831	0.048585
2	6	0	2.860363	0.318751	-0.013506
3	8	0	2.592764	1.497983	-0.024038
4	16	0	1.586887	-0.935930	-0.030073
5	6	0	0.135952	0.164624	-0.012409
6	1	0	4.599350	-0.181273	1.089094
7	1	0	4.315600	-1.265963	-0.290077
8	1	0	4.914965	0.400578	-0.558253
9	6	0	-1.098850	-0.725601	-0.013187
10	1	0	0.198464	0.787186	0.881949
11	1	0	0.187694	0.801426	-0.897233
12	7	0	-2.406609	0.034831	0.007108
13	1	0	-1.125988	-1.351459	-0.908780
14	1	0	-1.111996	-1.374324	0.866177
15	6	0	-2.526682	0.868152	1.246624
16	6	0	-2.537932	0.912652	-1.200030
17	6	0	-3.516655	-0.974692	-0.005495
18	1	0	-3.527686	1.298086	1.270497
19	1	0	-2.368479	0.228460	2.115259
20	1	0	-1.786956	1.666248	1.218298
21	1	0	-4.467911	-0.443188	0.011746
22	1	0	-3.434862	-1.572045	-0.913309
23	1	0	-3.422463	-1.607935	0.876508
24	1	0	-3.539855	1.341237	-1.201026
25	1	0	-1.799178	1.710620	-1.148142
26	1	0	-2.385026	0.305206	-2.092499

Structure:	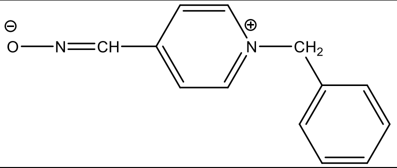
Geometry:	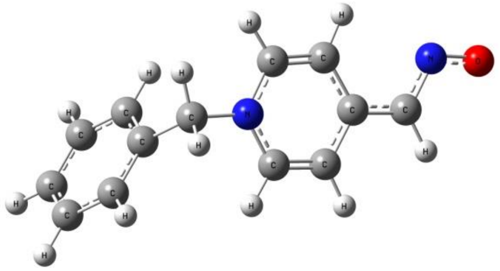
System:	**isolated reactant (IR):** reaction 1, nucleophile BPA4
(CPCM)/M06–2X/6–311++G(2df,2pd) Electronic Energy (in a.u.):	-687.19724
(CPCM)/M06–2X/6–31+G(d) Thermal Correction to Gibbs Free Energy (in a.u.)	0.184458
Number of imaginary frequencies:	0

CARTESIAN COORDINATES

Center Number	Atomic Number	Atomic Type	Coordinates (Angstroms) X	Y	Z
1	6	0	0.848414	-0.853425	1.269149
2	1	0	0.516986	-1.087165	2.274335
3	6	0	2.104813	-0.381383	1.006289
4	1	0	2.794824	-0.234578	1.827433
5	6	0	2.502456	-0.086889	-0.328318
6	6	0	1.520164	-0.314248	-1.332895
7	1	0	1.746570	-0.115990	-2.374810
8	6	0	0.278869	-0.784310	-1.006238
9	1	0	-0.493223	-0.961145	-1.747450
10	6	0	-1.424757	-1.537124	0.580984
11	1	0	-1.434535	-1.818188	1.636130
12	1	0	-1.602623	-2.439147	-0.009610
13	6	0	-2.465115	-0.481810	0.282629
14	6	0	-3.479012	-0.732703	-0.642312
15	1	0	-3.517460	-1.691327	-1.154055
16	6	0	-4.442907	0.241189	-0.912189
17	1	0	-5.228859	0.036865	-1.632939
18	6	0	-4.390807	1.472138	-0.261866
19	1	0	-5.137579	2.231647	-0.472961
20	6	0	-3.374694	1.728882	0.662395
21	1	0	-3.331226	2.686540	1.172288
22	6	0	-2.417784	0.755348	0.934607
23	1	0	-1.629221	0.958013	1.656517
24	6	0	3.786194	0.403519	-0.677628
25	1	0	4.034843	0.612497	-1.719601

26	7	0	-0.059727	-1.057449	0.280041
27	7	0	4.692272	0.609203	0.273440
28	8	0	5.822336	1.048076	-0.097161

Structure:	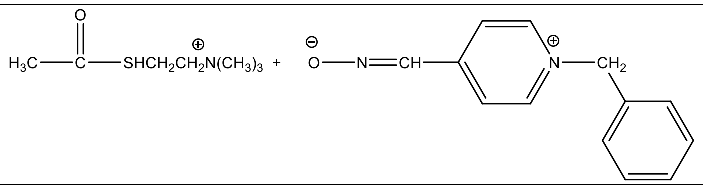
Geometry:	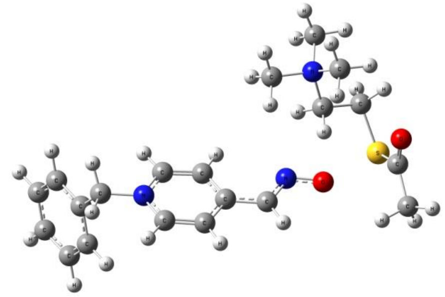
System:	**reactant complex (RC):** reaction 1, nucleophile BPA4
(CPCM)/M06–2X/6–311++G(2df,2pd) Electronic Energy (in a.u.):	-1491.55021
(CPCM)/M06–2X/6–31+G(d) Thermal Correction to Gibbs Free Energy (in a.u.)	0.398581
Number of imaginary frequencies:	0

CARTESIAN COORDINATES

Center Number	Atomic Number	Atomic Type	Coordinates (Angstroms) X	Y	Z
1	6	0	-5.000663	-2.860317	1.172189
2	6	0	-5.127135	-1.414067	0.787049
3	8	0	-5.391687	-1.025249	-0.326809
4	16	0	-4.813760	-0.263294	2.133144
5	6	0	-4.709393	1.248079	1.132070
6	1	0	-5.192306	-3.021346	2.235117
7	1	0	-5.682549	-3.459067	0.565294
8	1	0	-3.968664	-3.153326	0.947588
9	6	0	-3.346170	1.270116	0.443330
10	1	0	-5.543427	1.222437	0.428095
11	1	0	-4.845794	2.090321	1.812585
12	7	0	-3.286674	2.133155	-0.798551
13	1	0	-2.587315	1.658329	1.127729
14	1	0	-3.054128	0.265196	0.120982
15	6	0	-4.045316	1.478684	-1.911694
16	6	0	-3.829632	3.500845	-0.539719
17	6	0	-1.848356	2.240182	-1.204748
18	1	0	-3.953706	2.101635	-2.801637
19	1	0	-3.612956	0.491801	-2.081345
20	1	0	-5.091714	1.378408	-1.628966
21	1	0	-1.792187	2.782864	-2.148607
22	1	0	-1.307551	2.779592	-0.426384
23	1	0	-1.450541	1.229016	-1.310465
24	1	0	-3.670731	4.109963	-1.429915
25	1	0	-4.895756	3.430140	-0.326082
26	1	0	-3.299234	3.930107	0.311049

27	8	0	-2.364557	-1.660255	-0.300702
28	7	0	-1.196111	-1.164489	-0.406477
29	6	0	-0.243147	-1.957837	-0.868708
30	6	0	1.083311	-1.459027	-1.000987
31	6	0	2.111335	-2.279242	-1.530885
32	6	0	3.390310	-1.803746	-1.664170
33	7	0	3.714964	-0.542813	-1.294078
34	6	0	2.760474	0.272389	-0.771530
35	6	0	1.468206	-0.143521	-0.617373
36	1	0	1.898920	-3.296325	-1.841055
37	1	0	4.194991	-2.406855	-2.068352
38	6	0	5.100689	-0.040193	-1.419965
39	1	0	3.093231	1.263741	-0.482910
40	1	0	0.742220	0.536682	-0.189932
41	1	0	-0.477356	-2.986112	-1.149074
42	1	0	5.079290	0.848479	-2.055510
43	6	0	5.701392	0.273042	-0.069308
44	1	0	5.669796	-0.813820	-1.939340
45	6	0	5.821788	-0.733119	0.895611
46	6	0	6.375565	-0.447849	2.140397
47	6	0	6.817392	0.845937	2.429582
48	6	0	6.699715	1.850578	1.471549
49	6	0	6.138885	1.564565	0.224942
50	1	0	5.479066	-1.741587	0.672552
51	1	0	6.466166	-1.233524	2.884364
52	1	0	7.250670	1.067343	3.400329
53	1	0	7.038691	2.858268	1.691937
54	1	0	6.042258	2.350424	-0.520319

Structure:	
Geometry:	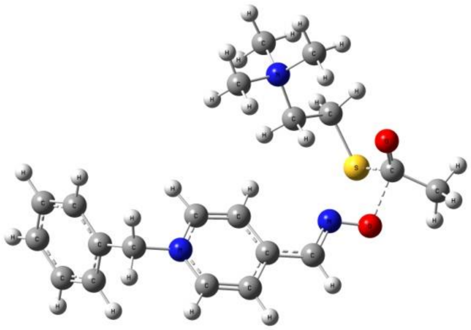
System:	**transition state 1 (TS1):** reaction 1, nucleophile BPA4
(CPCM)/M06–2X/6–311++G(2df,2pd) Electronic Energy (in a.u.):	-1491.53586
(CPCM)/M06–2X/6–31+G(d) Thermal Correction to Gibbs Free Energy (in a.u.)	0.403654
Number of imaginary frequencies:	1 (–198 cm^{-1})

CARTESIAN COORDINATES

Center Number	Atomic Number	Atomic Type	Coordinates (Angstroms) X	Y	Z
1	6	0	-5.407804	-2.138130	0.576176
2	6	0	-4.348963	-1.104358	0.262755
3	8	0	-4.285502	-0.517127	-0.814038
4	16	0	-3.852862	-0.146108	1.799684
5	6	0	-3.659535	1.481716	1.021602
6	1	0	-5.185967	-2.672694	1.502340
7	1	0	-6.367039	-1.621910	0.682552
8	1	0	-5.469092	-2.847764	-0.252619
9	6	0	-2.345186	1.544833	0.255563
10	1	0	-4.519124	1.635518	0.367123
11	1	0	-3.692115	2.214337	1.831175
12	7	0	-2.241376	2.720788	-0.698292
13	1	0	-1.505241	1.635220	0.950670
14	1	0	-2.224826	0.643406	-0.349491
15	6	0	-3.207122	2.564886	-1.833480
16	6	0	-2.502116	4.010221	0.013259
17	6	0	-0.853956	2.745760	-1.259670
18	1	0	-3.031736	3.374576	-2.542203
19	1	0	-3.035617	1.595996	-2.301861
20	1	0	-4.225536	2.619707	-1.454415
21	1	0	-0.772905	3.585508	-1.949913
22	1	0	-0.143607	2.862444	-0.440609
23	1	0	-0.676842	1.810616	-1.792147
24	1	0	-2.318274	4.830764	-0.680631

25	1	0	-3.539707	4.031773	0.343868
26	1	0	-1.828123	4.079719	0.867978
27	8	0	-2.952546	-2.433865	0.302516
28	7	0	-1.814001	-1.900244	0.017405
29	6	0	-0.820439	-2.729185	-0.139069
30	6	0	0.492626	-2.217143	-0.440797
31	6	0	1.557496	-3.098825	-0.710315
32	6	0	2.807269	-2.608047	-1.010551
33	7	0	3.045851	-1.280164	-1.047779
34	6	0	2.048538	-0.401871	-0.776953
35	6	0	0.785015	-0.834720	-0.474700
36	1	0	1.409038	-4.172653	-0.692008
37	1	0	3.648202	-3.254427	-1.231689
38	6	0	4.397015	-0.758511	-1.379147
39	1	0	2.316505	0.648781	-0.808879
40	1	0	0.011830	-0.112109	-0.249843
41	1	0	-0.973436	-3.805565	-0.051425
42	1	0	4.304776	-0.165911	-2.292162
43	6	0	4.965131	0.063498	-0.247615
44	1	0	5.020577	-1.627132	-1.596850
45	6	0	5.286650	-0.546426	0.969968
46	6	0	5.799670	0.211756	2.018652
47	6	0	5.999982	1.585056	1.856363
48	6	0	5.685042	2.195551	0.644264
49	6	0	5.165763	1.435465	-0.405587
50	1	0	5.136331	-1.616443	1.097766
51	1	0	6.047936	-0.267512	2.960692
52	1	0	6.402154	2.175140	2.674352
53	1	0	5.839717	3.262114	0.512851
54	1	0	4.918514	1.912351	-1.351032

Structure:	
Geometry:	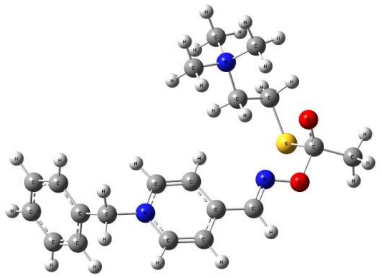
System:	**intermediate (IM):** reaction 1, nucleophile BPA4
(CPCM)/M06–2X/6–311++G(2df,2pd) Electronic Energy (in a.u.):	-1491.53903
(CPCM)/M06–2X/6–31+G(d) Thermal Correction to Gibbs Free Energy (in a.u.)	0.405777
Number of imaginary frequencies:	0

CARTESIAN COORDINATES

Center Number	Atomic Number	Atomic Type	Coordinates (Angstroms) X	Y	Z
1	6	0	-5.214488	-2.318129	0.589464
2	6	0	-4.042838	-1.422042	0.209331
3	8	0	-4.111566	-0.705051	-0.820633
4	16	0	-3.671166	-0.306646	1.895264
5	6	0	-3.789914	1.312112	1.084794
6	1	0	-5.033909	-2.858262	1.522461
7	1	0	-6.104603	-1.695583	0.701787
8	1	0	-5.381502	-3.037969	-0.220039
9	6	0	-2.509634	1.574386	0.306339
10	1	0	-4.653648	1.277976	0.417771
11	1	0	-3.958305	2.064350	1.859184
12	7	0	-2.587245	2.738614	-0.667476
13	1	0	-1.684077	1.799917	0.987944
14	1	0	-2.268392	0.695473	-0.295205
15	6	0	-3.590962	2.454592	-1.743317
16	6	0	-2.948030	4.003419	0.040941
17	6	0	-1.241666	2.897990	-1.301541
18	1	0	-3.493693	3.221735	-2.512072
19	1	0	-3.389751	1.462202	-2.147078
20	1	0	-4.592290	2.483013	-1.317936
21	1	0	-1.272476	3.748186	-1.983398
22	1	0	-0.502068	3.069934	-0.518874
23	1	0	-1.008468	1.986165	-1.852401
24	1	0	-2.929344	4.822255	-0.678778
25	1	0	-3.948198	3.903155	0.460028
26	1	0	-2.219880	4.179050	0.833797
27	8	0	-2.886921	-2.390611	0.239052
28	7	0	-1.725595	-1.804449	-0.076410
29	6	0	-0.754768	-2.636556	-0.159058
30	6	0	0.582727	-2.131714	-0.468516

31	6	0	1.632017	-3.027981	-0.700130
32	6	0	2.891887	-2.548250	-0.999609
33	7	0	3.128664	-1.225170	-1.064981
34	6	0	2.136875	-0.334211	-0.832972
35	6	0	0.864224	-0.756209	-0.535687
36	1	0	1.477300	-4.099968	-0.654101
37	1	0	3.733626	-3.201757	-1.193890
38	6	0	4.491654	-0.714613	-1.387054
39	1	0	2.411135	0.713283	-0.889900
40	1	0	0.088983	-0.025024	-0.345228
41	1	0	-0.900329	-3.707201	-0.010569
42	1	0	4.409339	-0.140792	-2.312525
43	6	0	5.041257	0.126831	-0.261699
44	1	0	5.112522	-1.590779	-1.578878
45	6	0	5.370165	-0.467082	0.961706
46	6	0	5.863948	0.310117	2.005657
47	6	0	6.037913	1.685479	1.831947
48	6	0	5.716661	2.279558	0.613290
49	6	0	5.216476	1.500938	-0.431990
50	1	0	5.241587	-1.538753	1.098086
51	1	0	6.117955	-0.156135	2.952610
52	1	0	6.425400	2.290232	2.646277
53	1	0	5.852180	3.347536	0.473060
54	1	0	4.965653	1.964611	-1.383007

Structure:	
Geometry:	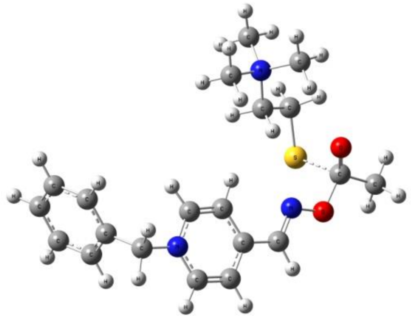
System:	**transition state 2 (TS2):** reaction 1, nucleophile BPA4
(CPCM)/M06–2X/6–311++G(2df,2pd) Electronic Energy (in a.u.):	-1491.53879
(CPCM)/M06–2X/6–31+G(d) Thermal Correction to Gibbs Free Energy (in a.u.)	0.404522
Number of imaginary frequencies:	1 (–154 cm^{-1})

CARTESIAN COORDINATES

Center Number	Atomic Number	Atomic Type	Coordinates (Angstroms) X	Y	Z
1	6	0	-5.168830	-2.382649	0.419969
2	6	0	-3.963974	-1.550060	0.032852
3	8	0	-3.978470	-0.775471	-0.930499
4	16	0	-3.670260	-0.328836	1.925111
5	6	0	-3.911739	1.256894	1.069586
6	1	0	-5.009238	-2.918182	1.357218
7	1	0	-6.031971	-1.722918	0.519880
8	1	0	-5.360820	-3.103725	-0.384231
9	6	0	-2.624847	1.619889	0.345613
10	1	0	-4.730977	1.128916	0.357089
11	1	0	-4.190796	2.019841	1.800625
12	7	0	-2.740595	2.789445	-0.622726
13	1	0	-1.845057	1.895165	1.061673
14	1	0	-2.291531	0.768232	-0.251121
15	6	0	-3.653317	2.441629	-1.757716
16	6	0	-3.244814	4.010344	0.075145
17	6	0	-1.379329	3.069324	-1.175093
18	1	0	-3.608333	3.246490	-2.492198
19	1	0	-3.320810	1.497649	-2.189528
20	1	0	-4.669944	2.338717	-1.383313
21	1	0	-1.445264	3.912134	-1.863517
22	1	0	-0.707367	3.307826	-0.350413
23	1	0	-1.031098	2.182279	-1.704571
24	1	0	-3.229166	4.843935	-0.627744
25	1	0	-4.264064	3.831366	0.414310
26	1	0	-2.593835	4.220369	0.924860

27	8	0	-2.823562	-2.446011	0.177339
28	7	0	-1.652764	-1.862816	-0.150229
29	6	0	-0.674771	-2.684600	-0.082834
30	6	0	0.675979	-2.205037	-0.386559
31	6	0	1.745269	-3.105717	-0.402565
32	6	0	3.018433	-2.654225	-0.692697
33	7	0	3.244355	-1.354255	-0.955241
34	6	0	2.230264	-0.458882	-0.938402
35	6	0	0.944018	-0.853732	-0.660655
36	1	0	1.598448	-4.159240	-0.193535
37	1	0	3.877781	-3.313025	-0.722126
38	6	0	4.620361	-0.869592	-1.261993
39	1	0	2.497679	0.570667	-1.147958
40	1	0	0.148954	-0.119214	-0.650235
41	1	0	-0.818866	-3.730386	0.191070
42	1	0	4.592992	-0.440271	-2.265817
43	6	0	5.084144	0.140294	-0.241129
44	1	0	5.262766	-1.751059	-1.280913
45	6	0	5.282193	-0.247376	1.088544
46	6	0	5.702870	0.684653	2.032889
47	6	0	5.934242	2.009258	1.653255
48	6	0	5.742387	2.397683	0.329138
49	6	0	5.314871	1.464215	-0.616911
50	1	0	5.107932	-1.279393	1.385835
51	1	0	5.855014	0.378403	3.063282
52	1	0	6.264521	2.734912	2.390343
53	1	0	5.921815	3.425551	0.029315
54	1	0	5.164198	1.767691	-1.650024

Structure:	
Geometry:	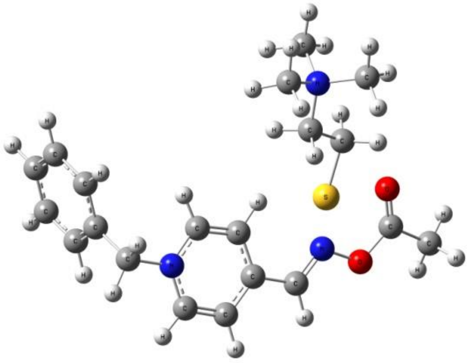
System:	**product complex (PC):** reaction 1, nucleophile BPA4
(CPCM)/M06–2X/6–311++G(2df,2pd) Electronic Energy (in a.u.):	-1491.54999
(CPCM)/M06–2X/6–31+G(d) Thermal Correction to Gibbs Free Energy (in a.u.)	0.401130
Number of imaginary frequencies:	0

CARTESIAN COORDINATES

Center Number	Atomic Number	Atomic Type	Coordinates (Angstroms) X	Y	Z
1	6	0	-5.107609	-2.490176	0.033719
2	6	0	-3.843683	-1.849013	-0.450816
3	8	0	-3.756039	-0.890437	-1.171828
4	16	0	-3.596443	0.027787	2.430926
5	6	0	-4.029638	1.274533	1.155619
6	1	0	-4.960260	-2.976514	0.997704
7	1	0	-5.881649	-1.725614	0.105990
8	1	0	-5.418469	-3.241278	-0.700374
9	6	0	-2.766376	1.648202	0.398750
10	1	0	-4.761564	0.849061	0.463136
11	1	0	-4.478215	2.154784	1.627114
12	7	0	-2.932051	2.722188	-0.676314
13	1	0	-2.003844	2.033271	1.081213
14	1	0	-2.375658	0.768462	-0.118192
15	6	0	-4.048253	2.375251	-1.608906
16	6	0	-3.194465	4.055524	-0.055599
17	6	0	-1.657572	2.792506	-1.454644
18	1	0	-4.023498	3.071952	-2.447627
19	1	0	-3.906052	1.349860	-1.950909
20	1	0	-4.995160	2.467446	-1.078806
21	1	0	-1.728417	3.608178	-2.175126
22	1	0	-0.834358	2.971061	-0.762030
23	1	0	-1.516514	1.843936	-1.973451
24	1	0	-3.288758	4.796547	-0.850257

25	1	0	-4.119373	4.007291	0.516872
26	1	0	-2.357852	4.306995	0.596786
27	8	0	-2.756830	-2.574626	-0.026179
28	7	0	-1.544771	-2.014101	-0.369057
29	6	0	-0.590122	-2.755518	0.033603
30	6	0	0.793310	-2.318356	-0.213310
31	6	0	1.849784	-3.164026	0.125582
32	6	0	3.151092	-2.756340	-0.107791
33	7	0	3.405509	-1.552585	-0.648663
34	6	0	2.399530	-0.711534	-0.978066
35	6	0	1.086353	-1.067386	-0.774683
36	1	0	1.676478	-4.138102	0.568402
37	1	0	4.006831	-3.375749	0.130970
38	6	0	4.813012	-1.118880	-0.899119
39	1	0	2.691372	0.243899	-1.398532
40	1	0	0.297006	-0.377264	-1.046583
41	1	0	-0.767156	-3.698615	0.551306
42	1	0	4.925304	-1.009363	-1.979861
43	6	0	5.126217	0.165987	-0.174259
44	1	0	5.451970	-1.937411	-0.565562
45	6	0	5.173627	0.187006	1.223974
46	6	0	5.453909	1.373793	1.894984
47	6	0	5.694771	2.545333	1.172792
48	6	0	5.653237	2.526591	-0.219753
49	6	0	5.366249	1.337858	-0.893027
50	1	0	4.992291	-0.725468	1.788186
51	1	0	5.489893	1.385172	2.979945
52	1	0	5.916324	3.469760	1.697450
53	1	0	5.841534	3.434190	-0.784831
54	1	0	5.332788	1.322771	-1.979741

Structure:	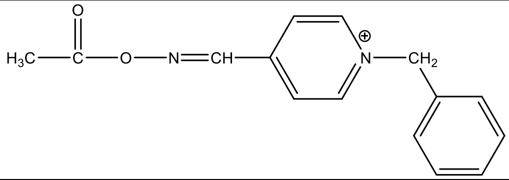
Geometry:	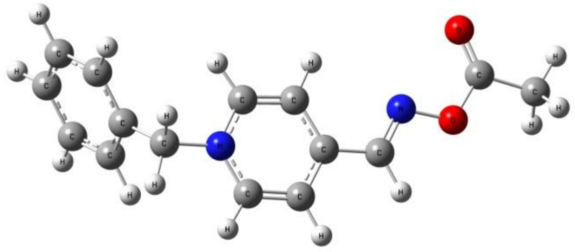
System:	**isolated product (IP)**: reaction 1, nucleophile BPA4
(CPCM)/M06–2X/6–311++G(2df,2pd) Electronic Energy (in a.u.):	-840.30821
(CPCM)/M06–2X/6–31+G(d) Thermal Correction to Gibbs Free Energy (in a.u.)	0.229310
Number of imaginary frequencies:	0

CARTESIAN COORDINATES

Center Number	Atomic Number	Atomic Type	Coordinates (Angstroms) X	Y	Z
1	6	0	0.545659	-0.017934	0.634024
2	6	0	1.216121	-1.039774	-0.053413
3	6	0	0.495103	-2.165902	-0.450413
4	6	0	-0.853714	-2.250555	-0.153905
5	7	0	-1.472895	-1.258211	0.507398
6	6	0	-0.797898	-0.153757	0.897114
7	6	0	2.653077	-0.957734	-0.363469
8	7	0	3.302190	0.067491	0.023531
9	8	0	4.627027	-0.040189	-0.352413
10	6	0	5.404419	1.030873	0.028838
11	1	0	3.127625	-1.772673	-0.910844
12	1	0	0.966756	-2.981462	-0.986474
13	1	0	-1.459773	-3.103092	-0.434712
14	1	0	-1.375292	0.601915	1.417141
15	1	0	1.067206	0.873636	0.960536
16	6	0	-2.926233	-1.362121	0.835407
17	1	0	-3.264292	-2.318704	0.435083
18	1	0	-3.003698	-1.393967	1.924106
19	6	0	-3.702968	-0.205831	0.257554
20	8	0	4.974917	1.978753	0.625964
21	6	0	6.815726	0.798131	-0.418411
22	1	0	7.203881	-0.111386	0.047583
23	1	0	6.843178	0.659868	-1.502169
24	1	0	7.426028	1.653539	-0.133498
25	6	0	-3.822399	-0.065800	-1.129444
26	6	0	-4.532591	1.005232	-1.663988

27	6	0	-5.133561	1.938847	-0.815724
28	6	0	-5.020886	1.799188	0.565930
29	6	0	-4.303141	0.728822	1.102466
30	1	0	-3.359991	-0.795162	-1.791345
31	1	0	-4.622404	1.110253	-2.740764
32	1	0	-5.689584	2.772342	-1.234100
33	1	0	-5.487385	2.521513	1.228607
34	1	0	-4.213157	0.619790	2.180547

Structure:	⊖ ⊕ $SHCH_2CH_2NH_3(CH_3)_3$
Geometry:	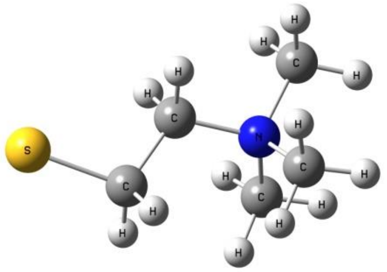
System:	**isolated product (IP)**: reaction 1, nucleophile BPA4
(CPCM)/M06–2X/6–311++G(2df,2pd) Electronic Energy (in a.u.):	-651.23314
(CPCM)/M06–2X/6–31+G(d) Thermal Correction to Gibbs Free Energy (in a.u.)	0.149700
Number of imaginary frequencies:	0

CARTESIAN COORDINATES

Center Number	Atomic Number	Atomic Type	Coordinates (Angstroms) X	Y	Z
1	16	0	2.893900	0.194179	-0.000032
2	6	0	1.231439	-0.587013	0.000121
3	6	0	0.180220	0.510361	-0.000464
4	1	0	1.120814	-1.220681	-0.884328
5	1	0	1.120636	-1.220004	0.885034
6	7	0	-1.274507	0.042496	-0.000027
7	1	0	0.286873	1.139257	0.887269
8	1	0	0.286739	1.138123	-0.889019
9	6	0	-1.571708	-0.768954	-1.219812
10	6	0	-1.571729	-0.766432	1.221416
11	6	0	-2.147269	1.257795	-0.001219
12	1	0	-2.638720	-0.993459	-1.235198
13	1	0	-1.295282	-0.188338	-2.100653
14	1	0	-1.000224	-1.694651	-1.181921
15	1	0	-3.190980	0.942714	-0.000437
16	1	0	-1.930747	1.843701	0.892156
17	1	0	-1.931384	1.841535	-0.896158
18	1	0	-2.638639	-0.991413	1.236914
19	1	0	-0.999775	-1.691917	1.185734
20	1	0	-1.295878	-0.183746	2.101063

Structure:	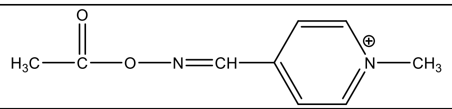
Geometry:	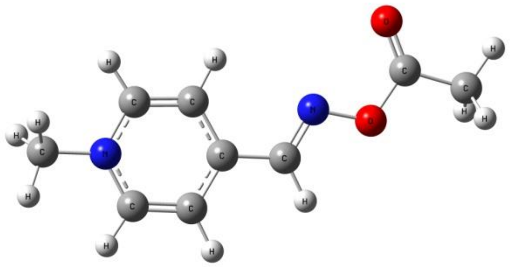
System:	**isolated reactant (IR)**: reaction 2, nucleophile HO^{-}
(CPCM)/M06–2X/6–311++G(2df,2pd) Electronic Energy (in a.u.):	-609.27984
(CPCM)/M06–2X/6–31+G(d) Thermal Correction to Gibbs Free Energy (in a.u.)	0.154211
Number of imaginary frequencies:	0

CARTESIAN COORDINATES

Center Number	Atomic Number	Atomic Type	Coordinates (Angstroms) X	Y	Z
1	6	0	1.196332	-0.864408	-0.057966
2	6	0	0.813359	0.483353	-0.027291
3	6	0	1.807029	1.461288	0.020258
4	6	0	3.136867	1.081512	0.042032
5	7	0	3.478888	-0.218243	0.015950
6	6	0	2.534847	-1.184430	-0.035198
7	6	0	-0.601381	0.891753	-0.038787
8	7	0	-1.495737	-0.014533	-0.013818
9	8	0	-2.753280	0.556796	-0.024992
10	6	0	-3.781731	-0.358145	0.020620
11	1	0	-0.849883	1.953208	-0.062372
12	1	0	1.562556	2.517064	0.044270
13	1	0	3.947548	1.798364	0.079701
14	1	0	2.895990	-2.205805	-0.057334
15	1	0	0.458356	-1.656448	-0.097682
16	6	0	4.902054	-0.617470	0.022045
17	1	0	5.511331	0.259845	0.225216
18	1	0	5.050512	-1.363515	0.801583
19	1	0	5.151730	-1.031993	-0.954724
20	8	0	-3.608435	-1.544549	0.067178
21	6	0	-5.090586	0.371796	0.001463
22	1	0	-5.140366	1.065651	0.844396
23	1	0	-5.173854	0.954172	-0.919888
24	1	0	-5.902988	-0.350577	0.061362

Structure:	HO^-
Geometry:	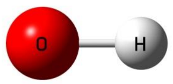
System:	**isolated reactant (IR)**: reaction 2, nucleophile HO^-
(CPCM)/M06–2X/6–311++G(2df,2pd) Electronic Energy (in a.u.):	-75.92012
(CPCM)/M06–2X/6–31+G(d) Thermal Correction to Gibbs Free Energy (in a.u.)	-0.007619
Number of imaginary frequencies:	0

CARTESIAN COORDINATES

Center Number	Atomic Number	Atomic Type	Coordinates (Angstroms) X	Y	Z
1	8	0	0.000000	0.000000	0.107644
2	1	0	0.000000	0.000000	-0.861152

Structure:	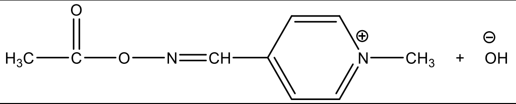
Geometry:	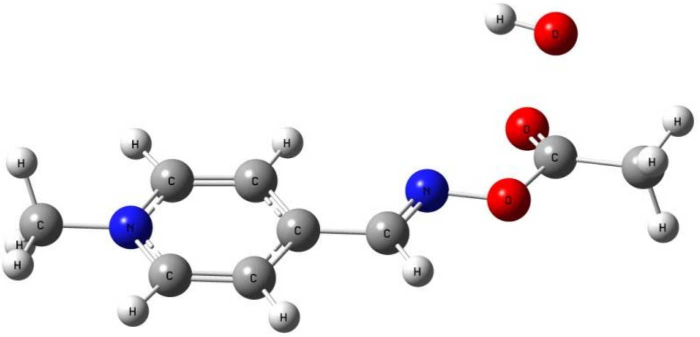
System:	**reactant complex (RC)**: reaction 2, nucleophile HO^{-}
(CPCM)/M06–2X/6–311++G(2df,2pd) Electronic Energy (in a.u.):	-685.20387
(CPCM)/M06–2X/6–31+G(d) Thermal Correction to Gibbs Free Energy (in a.u.)	0.162883
Number of imaginary frequencies:	0

CARTESIAN COORDINATES

Center Number	Atomic Number	Atomic Type	Coordinates (Angstroms) X	Y	Z
1	6	0	1.504513	-0.811324	-0.216026
2	6	0	1.151585	0.532588	-0.024363
3	6	0	2.168839	1.464574	0.188077
4	6	0	3.486768	1.047205	0.203494
5	7	0	3.798624	-0.247222	0.017201
6	6	0	2.832412	-1.170117	-0.190797
7	6	0	-0.248179	0.981818	-0.038583
8	7	0	-1.163266	0.123729	-0.266243
9	8	0	-2.398745	0.709370	-0.225033
10	6	0	-3.439885	-0.165985	-0.504896
11	1	0	-0.471734	2.033516	0.143532
12	1	0	1.949822	2.514814	0.342755
13	1	0	4.312124	1.729147	0.364639
14	1	0	3.168787	-2.190479	-0.332087
15	1	0	0.748757	-1.569450	-0.382051
16	6	0	5.207593	-0.691042	0.036490
17	1	0	5.844189	0.168772	0.231147
18	1	0	5.328666	-1.432334	0.825880
19	1	0	5.450396	-1.124485	-0.933431
20	8	0	-3.258781	-1.246777	-1.000812
21	6	0	-4.741844	0.555189	-0.350574
22	1	0	-4.730908	1.170484	0.547160
23	1	0	-4.898973	1.189164	-1.230711
24	1	0	-5.542532	-0.180839	-0.290350
25	8	0	-3.629157	-0.781544	1.963633
26	1	0	-2.843459	-1.349703	1.937568

Structure:	
Geometry:	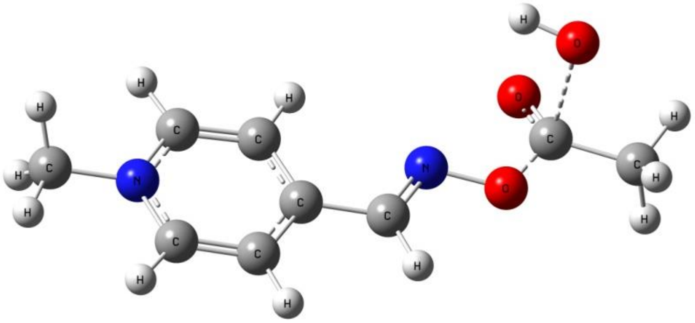
System:	**transition state 1 (TS1)**: reaction 2, nucleophile HO^{-}
(CPCM)/M06–2X/6–311++G(2df,2pd) Electronic Energy (in a.u.):	-685.20356
(CPCM)/M06–2X/6–31+G(d) Thermal Correction to Gibbs Free Energy (in a.u.)	0.163530
Number of imaginary frequencies:	1 (–117 cm^{-1})

CARTESIAN COORDINATES

Center Number	Atomic Number	Atomic Type	Coordinates (Angstroms) X	Y	Z
1	6	0	1.502440	-0.813914	-0.205016
2	6	0	1.149366	0.534137	-0.042750
3	6	0	2.167441	1.469215	0.153737
4	6	0	3.484741	1.051232	0.182873
5	7	0	3.796481	-0.247036	0.023568
6	6	0	2.829773	-1.173125	-0.168199
7	6	0	-0.249171	0.984169	-0.073324
8	7	0	-1.167461	0.116744	-0.249600
9	8	0	-2.400323	0.701239	-0.238710
10	6	0	-3.446080	-0.200160	-0.446703
11	1	0	-0.469929	2.044499	0.053951
12	1	0	1.948797	2.522629	0.285772
13	1	0	4.309933	1.735868	0.333470
14	1	0	3.165677	-2.196307	-0.288835
15	1	0	0.746629	-1.574478	-0.359192
16	6	0	5.205135	-0.691254	0.050033
17	1	0	5.841491	0.168401	0.246046
18	1	0	5.322657	-1.431267	0.841096
19	1	0	5.452142	-1.126463	-0.918109
20	8	0	-3.262969	-1.292784	-0.921148
21	6	0	-4.746795	0.536413	-0.355506
22	1	0	-4.735273	1.242238	0.472870
23	1	0	-4.906878	1.076751	-1.295928
24	1	0	-5.549220	-0.187788	-0.219092
25	8	0	-3.598216	-0.692593	1.904540
26	1	0	-2.818185	-1.269206	1.901856

Structure:	
Geometry:	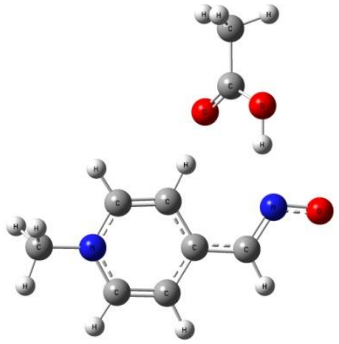
System:	**product complex (PC)**: reaction 2, nucleophile HO^{-}
(CPCM)/M06–2X/6–311++G(2df,2pd) Electronic Energy (in a.u.):	-685.27002
(CPCM)/M06–2X/6–31+G(d) Thermal Correction to Gibbs Free Energy (in a.u.)	0.161708
Number of imaginary frequencies:	0

CARTESIAN COORDINATES

Center Number	Atomic Number	Atomic Type	Coordinates (Angstroms) X	Y	Z
1	6	0	-0.731292	-0.262579	-0.288918
2	6	0	-1.013629	1.094288	0.019275
3	6	0	-2.369680	1.405097	0.280427
4	6	0	-3.336551	0.432938	0.229281
5	7	0	-3.028337	-0.848891	-0.070745
6	6	0	-1.739179	-1.187637	-0.325681
7	6	0	-0.027524	2.125689	0.074988
8	7	0	1.241380	1.862092	-0.169238
9	8	0	2.081406	2.814957	-0.110244
10	6	0	2.945514	-1.115855	0.213468
11	1	0	-0.317913	3.147725	0.318603
12	1	0	-2.664800	2.419483	0.524570
13	1	0	-4.381453	0.642205	0.424012
14	1	0	-1.562000	-2.231851	-0.557049
15	1	0	0.279126	-0.592462	-0.492502
16	6	0	-4.067774	-1.888603	-0.120795
17	1	0	-5.031638	-1.431544	0.093963
18	1	0	-4.085062	-2.331585	-1.117077
19	1	0	-3.848506	-2.652589	0.625926
20	8	0	2.276194	-1.077472	1.232681
21	6	0	3.968175	-2.177273	-0.089535
22	1	0	4.944738	-1.711479	-0.247022
23	1	0	4.023598	-2.889706	0.732503
24	1	0	3.695374	-2.694605	-1.013566
25	8	0	2.834540	-0.215121	-0.755625
26	1	0	2.151769	0.498701	-0.502037

Structure:	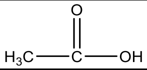
Geometry:	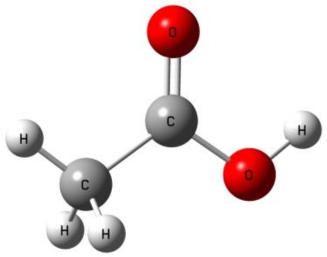
System:	**isolated product (IP)**: reaction 2, nucleophile HO^-
(CPCM)/M06–2X/6–311++G(2df,2pd) Electronic Energy (in a.u.):	-229.08610
(CPCM)/M06–2X/6–31+G(d) Thermal Correction to Gibbs Free Energy (in a.u.)	0.035191
Number of imaginary frequencies:	0

CARTESIAN COORDINATES

Center	Atomic	Atomic	Coordinates (Angstroms)		
Number	Number	Type	X	Y	Z
1	1	0	-1.735402	-0.818564	0.000189
2	8	0	-0.783878	-1.031057	0.000056
3	6	0	-0.088999	0.120186	-0.000125
4	8	0	-0.633705	1.202725	-0.000016
5	6	0	1.392265	-0.118402	0.000001
6	1	0	1.669039	-0.699366	0.883723
7	1	0	1.669042	-0.700870	-0.882718
8	1	0	1.918391	0.834749	-0.000764

Structure:	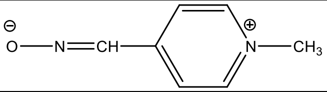
Geometry:	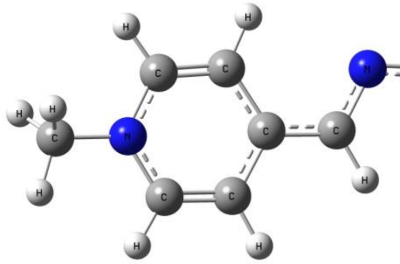
System:	**isolated product (IP)**: reaction 2, nucleophile HO^{-}
(CPCM)/M06–2X/6–311++G(2df,2pd) Electronic Energy (in a.u.):	-456.16824
(CPCM)/M06–2X/6–31+G(d) Thermal Correction to Gibbs Free Energy (in a.u.)	0.109491
Number of imaginary frequencies:	0

CARTESIAN COORDINATES

Center Number	Atomic Number	Atomic Type	Coordinates (Angstroms) X	Y	Z
1	6	0	0.046939	-1.007066	-0.000304
2	6	0	0.624561	0.295273	-0.000119
3	6	0	-0.292565	1.379133	-0.000150
4	6	0	-1.644456	1.156869	-0.000220
5	7	0	-2.154522	-0.097485	-0.000364
6	6	0	-1.310226	-1.163494	-0.000394
7	6	0	2.024211	0.532743	0.000054
8	7	0	2.864719	-0.495218	0.000122
9	8	0	4.104757	-0.223817	0.000317
10	1	0	2.412236	1.552994	0.000157
11	1	0	0.066839	2.402310	-0.000143
12	1	0	-2.365751	1.965402	-0.000211
13	1	0	-1.784847	-2.138369	-0.000503
14	1	0	0.680545	-1.885017	-0.000412
15	6	0	-3.605060	-0.328892	0.000722
16	1	0	-4.113299	0.633479	-0.004414
17	1	0	-3.882643	-0.893955	-0.890063
18	1	0	-3.882942	-0.884774	0.897216

Structure:	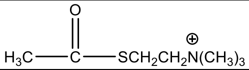
Geometry:	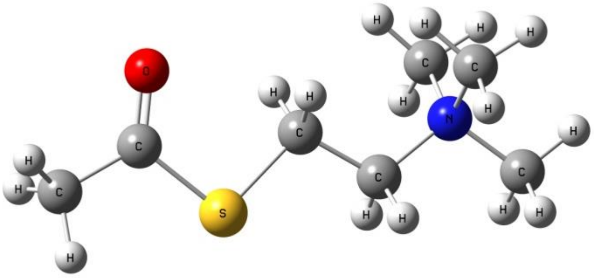
System:	**isolated reactant (IR)**: reaction 3, nucleophile HO^{-}
(CPCM)/M06–2X/6–311++G(2df,2pd) Electronic Energy (in a.u.):	-804.33933
(CPCM)/M06–2X/6–31+G(d) Thermal Correction to Gibbs Free Energy (in a.u.)	0.193617
Number of imaginary frequencies:	0

CARTESIAN COORDINATES

Center Number	Atomic Number	Atomic Type	Coordinates (Angstroms) X	Y	Z
1	6	0	4.261853	-0.229831	0.048585
2	6	0	2.860363	0.318751	-0.013506
3	8	0	2.592764	1.497983	-0.024038
4	16	0	1.586887	-0.935930	-0.030073
5	6	0	0.135952	0.164624	-0.012409
6	1	0	4.599350	-0.181273	1.089094
7	1	0	4.315600	-1.265963	-0.290077
8	1	0	4.914965	0.400578	-0.558253
9	6	0	-1.098850	-0.725601	-0.013187
10	1	0	0.198464	0.787186	0.881949
11	1	0	0.187694	0.801426	-0.897233
12	7	0	-2.406609	0.034831	0.007108
13	1	0	-1.125988	-1.351459	-0.908780
14	1	0	-1.111996	-1.374324	0.866177
15	6	0	-2.526682	0.868152	1.246624
16	6	0	-2.537932	0.912652	-1.200030
17	6	0	-3.516655	-0.974692	-0.005495
18	1	0	-3.527686	1.298086	1.270497
19	1	0	-2.368479	0.228460	2.115259
20	1	0	-1.786956	1.666248	1.218298
21	1	0	-4.467911	-0.443188	0.011746
22	1	0	-3.434862	-1.572045	-0.913309
23	1	0	-3.422463	-1.607935	0.876508
24	1	0	-3.539855	1.341237	-1.201026
25	1	0	-1.799178	1.710620	-1.148142
26	1	0	-2.385026	0.305206	-2.092499

Structure:	HO^-
Geometry:	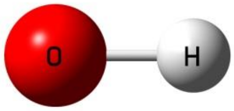
System:	**isolated reactant (IR)**: reaction 2, nucleophile HO^-
(CPCM)/M06–2X/6–311++G(2df,2pd) Electronic Energy (in a.u.):	-75.92012
(CPCM)/M06–2X/6–31+G(d) Thermal Correction to Gibbs Free Energy (in a.u.)	-0.007619
Number of imaginary frequencies:	0

CARTESIAN COORDINATES

Center Number	Atomic Number	Atomic Type	Coordinates (Angstroms) X	Y	Z
1	8	0	0.000000	0.000000	0.107644
2	1	0	0.000000	0.000000	-0.861152

Structure:	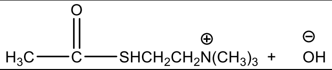
Geometry:	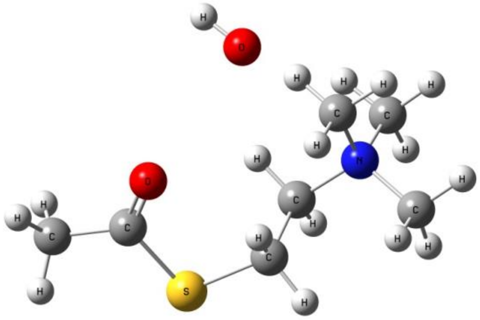
System:	**reactant complex (RC)**: reaction 3, nucleophile HO^{-}
(CPCM)/M06–2X/6–311++G(2df,2pd) Electronic Energy (in a.u.):	-880.27825
(CPCM)/M06–2X/6–31+G(d) Thermal Correction to Gibbs Free Energy (in a.u.)	0.202878
Number of imaginary frequencies:	0

CARTESIAN COORDINATES

Center Number	Atomic Number	Atomic Type	Coordinates (Angstroms) X	Y	Z
1	6	0	-3.861318	0.666313	-0.110436
2	6	0	-2.497686	0.204277	0.330795
3	8	0	-1.852489	0.735004	1.205548
4	16	0	-1.879514	-1.206851	-0.583686
5	6	0	-0.221125	-1.318977	0.145655
6	1	0	-3.727410	1.434638	-0.879492
7	1	0	-4.450919	-0.148709	-0.534857
8	1	0	-4.381469	1.108437	0.741198
9	6	0	0.721726	-0.371673	-0.587785
10	1	0	-0.322910	-1.077937	1.205933
11	1	0	0.078043	-2.363815	0.042485
12	7	0	2.083140	-0.240508	0.062429
13	1	0	0.900526	-0.741386	-1.601339
14	1	0	0.339885	0.656222	-0.638072
15	6	0	1.966011	0.552188	1.329977
16	6	0	2.692817	-1.573751	0.336254
17	6	0	2.956895	0.526346	-0.886596
18	1	0	2.966424	0.663665	1.751152
19	1	0	1.534008	1.520427	1.055518
20	1	0	1.333385	0.010887	2.033227
21	1	0	3.926667	0.675257	-0.409064
22	1	0	3.077087	-0.065332	-1.795400
23	1	0	2.455271	1.480815	-1.080495
24	1	0	3.714528	-1.418997	0.684045
25	1	0	2.117391	-2.084683	1.107944
26	1	0	2.695284	-2.155863	-0.586703
27	8	0	0.883530	2.740628	-0.557151
28	1	0	0.442197	3.586151	-0.728486

Structure:	
Geometry:	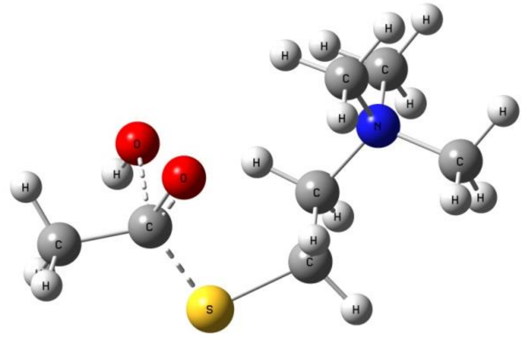
System:	**transition state 1 (TS1)**: reaction 3, nucleophile HO^{-}
(CPCM)/M06–2X/6–311++G(2df,2pd) Electronic Energy (in a.u.):	-880.26836
(CPCM)/M06–2X/6–31+G(d) Thermal Correction to Gibbs Free Energy (in a.u.)	0.205042
Number of imaginary frequencies:	1 (–246 cm^{-1})

CARTESIAN COORDINATES

Center Number	Atomic Number	Atomic Type	Coordinates (Angstroms) X	Y	Z
1	6	0	3.494356	-0.550271	0.266954
2	6	0	2.056813	-0.149257	0.462584
3	8	0	1.372913	-0.479082	1.410013
4	16	0	1.601041	1.340233	-0.510830
5	6	0	-0.133765	1.418865	0.016172
6	1	0	3.613643	-1.588975	0.576079
7	1	0	3.803641	-0.436917	-0.772795
8	1	0	4.117403	0.093143	0.900821
9	6	0	-0.896186	0.298677	-0.686917
10	1	0	-0.140688	1.313724	1.102932
11	1	0	-0.507827	2.410831	-0.245824
12	7	0	-2.154305	-0.146652	0.030797
13	1	0	-1.215147	0.629251	-1.679664
14	1	0	-0.252392	-0.591993	-0.790933
15	6	0	-1.801652	-0.860018	1.298882
16	6	0	-3.050239	1.010651	0.327220
17	6	0	-2.869621	-1.105852	-0.867704
18	1	0	-2.721303	-1.238675	1.745891
19	1	0	-1.120478	-1.674258	1.052251
20	1	0	-1.309097	-0.168566	1.979578
21	1	0	-3.753810	-1.478902	-0.350027
22	1	0	-3.158827	-0.582001	-1.778890
23	1	0	-2.191186	-1.926607	-1.102757
24	1	0	-3.973867	0.633710	0.767747
25	1	0	-2.550521	1.677600	1.029173
26	1	0	-3.262674	1.533442	-0.606205
27	8	0	1.327844	-1.747046	-1.018188
28	1	0	1.682313	-1.589712	-1.907420

Structure:	
Geometry:	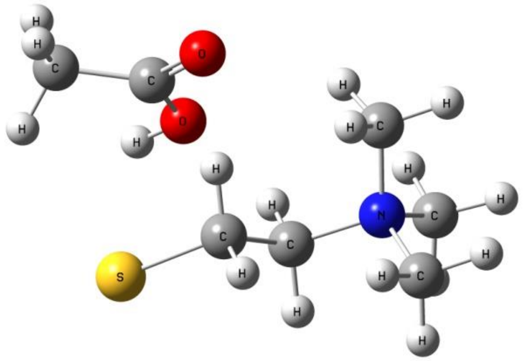
System:	**product complex (PC)**: reaction 3, nucleophile HO^{-}
(CPCM)/M06–2X/6–311++G(2df,2pd) Electronic Energy (in a.u.):	-880.32360
(CPCM)/M06–2X/6–31+G(d) Thermal Correction to Gibbs Free Energy (in a.u.)	0.205981
Number of imaginary frequencies:	0

CARTESIAN COORDINATES

Center Number	Atomic Number	Atomic Type	Coordinates (Angstroms) X	Y	Z
1	6	0	-3.618105	-0.459789	-0.378175
2	6	0	-2.254195	-1.036352	-0.115461
3	8	0	-1.432347	-1.292096	-0.967534
4	16	0	-1.096275	2.278713	0.256713
5	6	0	0.316554	1.403909	-0.525395
6	1	0	-4.373082	-1.235911	-0.212768
7	1	0	-3.818712	0.376090	0.295695
8	1	0	-3.671314	-0.116722	-1.410562
9	6	0	0.926875	0.464904	0.500417
10	1	0	-0.041378	0.829207	-1.383532
11	1	0	1.056803	2.127933	-0.880412
12	7	0	2.143491	-0.331053	0.030381
13	1	0	1.263381	1.018632	1.381168
14	1	0	0.189218	-0.279591	0.807785
15	6	0	1.842954	-1.059084	-1.241342
16	6	0	3.319018	0.568445	-0.170497
17	6	0	2.471469	-1.330739	1.092769
18	1	0	2.662844	-1.749605	-1.443408
19	1	0	0.899893	-1.593100	-1.117317
20	1	0	1.759634	-0.336356	-2.051964
21	1	0	3.374273	-1.869793	0.803775
22	1	0	2.632209	-0.802237	2.032892
23	1	0	1.633782	-2.022359	1.188283
24	1	0	4.171743	-0.036407	-0.480973
25	1	0	3.082170	1.297948	-0.943370
26	1	0	3.537835	1.073055	0.771121
27	8	0	-1.964818	-1.341710	1.165287
28	1	0	-2.673427	-1.060136	1.767591

Structure:	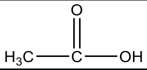
Geometry:	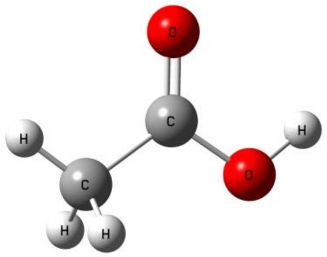
System:	**isolated product (IP)**: reaction 3, nucleophile HO^{-}
(CPCM)/M06–2X/6–311++G(2df,2pd) Electronic Energy (in a.u.):	-229.08610
(CPCM)/M06–2X/6–31+G(d) Thermal Correction to Gibbs Free Energy (in a.u.)	0.035191
Number of imaginary frequencies:	0

CARTESIAN COORDINATES

Center Number	Atomic Number	Atomic Type	Coordinates (Angstroms) X	Y	Z
1	1	0	-1.735402	-0.818564	0.000189
2	8	0	-0.783878	-1.031057	0.000056
3	6	0	-0.088999	0.120186	-0.000125
4	8	0	-0.633705	1.202725	-0.000016
5	6	0	1.392265	-0.118402	0.000001
6	1	0	1.669039	-0.699366	0.883723
7	1	0	1.669042	-0.700870	-0.882718
8	1	0	1.918391	0.834749	-0.000764

Structure:	$\overset{\ominus}{S}HCH_2CH_2\overset{\oplus}{N}H(CH_3)_3$
Geometry:	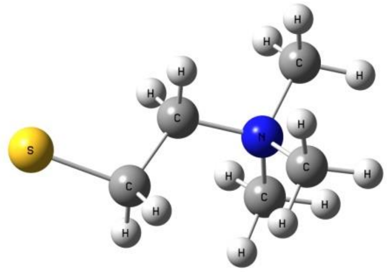
System:	**isolated product (IP)**: reaction 3, nucleophile HO^-
(CPCM)/M06–2X/6–311++G(2df,2pd) Electronic Energy (in a.u.):	-651.23314
(CPCM)/M06–2X/6–31+G(d) Thermal Correction to Gibbs Free Energy (in a.u.)	0.149700
Number of imaginary frequencies:	0

CARTESIAN COORDINATES

Center Number	Atomic Number	Atomic Type	Coordinates (Angstroms) X	Y	Z
1	16	0	2.893900	0.194179	-0.000032
2	6	0	1.231439	-0.587013	0.000121
3	6	0	0.180220	0.510361	-0.000464
4	1	0	1.120814	-1.220681	-0.884328
5	1	0	1.120636	-1.220004	0.885034
6	7	0	-1.274507	0.042496	-0.000027
7	1	0	0.286873	1.139257	0.887269
8	1	0	0.286739	1.138123	-0.889019
9	6	0	-1.571708	-0.768954	-1.219812
10	6	0	-1.571729	-0.766432	1.221416
11	6	0	-2.147269	1.257795	-0.001219
12	1	0	-2.638720	-0.993459	-1.235198
13	1	0	-1.295282	-0.188338	-2.100653
14	1	0	-1.000224	-1.694651	-1.181921
15	1	0	-3.190980	0.942714	-0.000437
16	1	0	-1.930747	1.843701	0.892156
17	1	0	-1.931384	1.841535	-0.896158
18	1	0	-2.638639	-0.991413	1.236914
19	1	0	-0.999775	-1.691917	1.185734
20	1	0	-1.295878	-0.183746	2.101063
